# Association between joint tenderness, patient-reported joint pain and ultrasound abnormalities in anti-CCP positive individuals at risk of rheumatoid arthritis: a cross-sectional study from a Leeds (UK) cohort

**DOI:** 10.1136/bmjopen-2026-117366

**Published:** 2026-06-04

**Authors:** Leticia Garcia-Montoya, Jing Kang, Laurence Duquenne, Andrea Di Matteo, Kate Harnden, Jacqueline L. Nam, Rahaymin Chowdhury, Richard J Wakefield, Kulveer S. Mankia, Paul Emery

**Affiliations:** 1 Institute of Rheumatic and Musculoskeletal Medicine, University of Leeds Leeds, Leeds, UK; 2 NIHR Leeds Musculoskeletal Biomedical Research Unit, Leeds, UK; 3 University of Leeds School of Dentistry, Leeds, UK; 4 Faculty of Densistry, Oral & Craniofacial Sciences, King’s College London, London, UK

**Keywords:** Ultrasonography, Risk Factors, RHEUMATOLOGY, Patient Reported Outcome Measures, Physical Examination

## Abstract

**Objectives:**

In individuals at-risk of rheumatoid arthritis (RA), to investigate how joint tenderness and patient-reported joint pain (PRJP) relate to ultrasound abnormalities and assess whether these exploratory results could be used to assist future evaluation of symptom/signs-guided ultrasound scanning approaches in this population.

**Methods:**

This is a cross-sectional analysis from a Leeds (UK) cohort of anti-cyclic citrullinated peptide (anti-CCP) positive individuals with new musculoskeletal complaints and no clinical arthritis. Assessments included physical examination, a mannequin where participants ticked joints that were painful and ultrasound scans of wrists, metacarpo-phalangeal joints 1–5 (MCPs1-5), proximal interphalangeal joints 1–5 (PIPs1-5), elbows, knees, ankles, metatarso-phalangeal joints 1–5 (MTPs1-5), finger flexor tendons (2-5) and extensor carpi ulnaris. Grey scale (GS), power Doppler (PD), tenosynovitis and erosions were assessed. A generalised estimating equations model was used to evaluate potential associations between tenderness/PRJP and ultrasound findings at the joint-level, adjusting for age and sex. Positive and negative predictive values for ultrasound changes were calculated.

**Results:**

323 participants were analysed. Joint tenderness was associated with ultrasound abnormalities, predominantly PD in wrists, MCPs, PIPs, elbows, knees and MTPs. GS and erosions were also associated with tenderness, but to a lesser degree. Association of PRJP with ultrasound abnormalities was more inconsistent and mostly for GS in the feet (all p*≤*0.05). Absence of symptoms and signs had a negative predictive value between 97% and 100% in all joints, except in wrists; which was slightly lower.

**Conclusions:**

In anti-CCP positive individuals at risk of RA, tenderness, predominantly in the small joints, was associated with local inflammatory changes on ultrasound. The association of PRJP and ultrasound was limited. In the absence of tenderness, the presence of PD, tenosynovitis or erosions was uncommon. These findings may inform future studies evaluating symptom/sign-guided ultrasound assessment approaches in at-risk populations.

**Trial registration number:**

NCT02012764.

**STRENGTHS AND LIMITATIONS OF THIS STUDY**

- Joint-level analyses accounted for clustering of multiple joints within the same participant in individuals at risk of rheumatoid arthritis.
- Well-characterised cohort of anti-cyclic citrullinated peptide-positive individuals assessed using standardised and validated European Alliance of Associations for Rheumatology (EULAR)/Outcome Measures in Rheumatology (OMERACT) definitions and scoring systems.
- Same-day data acquisition of clinical, patient and imaging data, reducing temporal variability between symptoms, signs and imaging findings.
- Due to the cross-sectional design of the study, causal relationships cannot be determined.
- Dichotomisation of ultrasound scores may have reduced sensitivity to the inflammatory burden.

## Introduction

 Rheumatoid arthritis (RA) is a disease continuum, with patients going through different phases before developing clinical arthritis.^1^ From the moment autoimmunity appears, patients may experience symptoms which can be secondary to inflammation. Initially, individuals have no joint involvement, but when this appears and is not evident on physical examination, it is called subclinical synovitis. This requires the use of sensitive imaging such as ultrasound in order to detect it.^2^ Even though not all subjects with subclinical synovitis develop RA, studies have reported its predictive role for disease progression, especially when there is significant power Doppler (PD) on ultrasound.^3 4^

Despite being a relatively ‘quick’ test, assessing the presence of subclinical synovitis on ultrasound usually involves the systematic performance of multiple joint scans, which is time-consuming and therefore rarely feasible to do in routine clinical appointments. Availability of ultrasound machines to use on demand in Rheumatology outpatients also varies across regions, which means that individuals may be referred to dedicated ultrasound clinics (ie, either Radiology or Rheumatology musculoskeletal ultrasound clinic) with the corresponding waiting times, which sometimes results in delayed clinical decisions.

Studies have proposed reduced scanning protocols for patients with established RA, especially in those who are in clinical remission.^5^ However, there is a scarcity of data for such protocols in patients at-risk of RA,^6^ where subclinical synovitis is prevalent and with correct interpretation, predictive of progression to clinical disease.^3 4^ Most importantly, to date, no studies have addressed the potential association between symptoms/signs and ultrasound findings in at-risk individuals.

Considering these factors, the objective of this exploratory study was to evaluate how clinical information, in particular joint symptoms (patient-reported joint pain (PRJP)) and signs (joint tenderness on palpation) correspond with ultrasound abnormalities at the joint level (focusing on the specific location) and whether these associations could be used to assist future evaluation of symptom/signs-guided ultrasound scanning approaches in individuals at-risk of RA.

## Methods

### Study design and participants

This is a cross-sectional analysis of the baseline data from the CCP Study (Coordinated Programme to Prevent Arthritis—can we identify Arthritis at a Pre-clinical Stage?) (trial registration number: NCT02012764), a prospective, observational cohort of individuals at-risk of RA which was adopted by the National Institute for Health and Care Research, Clinical Research Network.

Participants were recruited by primary care and secondary care between June 2008 and February 2020. However, this was not limited to doctors; other health professionals (ie, nurses, physiotherapists and musculoskeletal physicians) could also refer potential candidates. Participants gave written informed consent for the collection and the publication of their anonymised data.

Eligible candidates had to be ≥18 years old, have a new non-specific musculoskeletal complaint and no history of inflammatory arthritis. Either current or previous treatment with disease-modifying antirheumatic drugs was not allowed.

Primary care candidates were recruited across England for part 1 of the CCP study; which involved testing for the presence of anti-cyclic citrullinated peptide (anti-CCP antibodies. Those with a positive result were invited to attend Leeds to participate in part 2 of the study. Secondary care participants were recruited in Leeds and were required to have a positive anti-CCP antibodies test prior to referral to part 2 of the study.

Part 2 of the CCP study, which included the baseline assessments used in this cross-sectional analysis, took place in a dedicated Rheumatology clinic at Chapel Allerton Hospital in Leeds (UK). Participants with a diagnosis of fibromyalgia at baseline were excluded from this analysis.

### Assessments

This project analysed data from the baseline assessments of the secondary care part of the CCP Study, which consisted of: (1) collection of demographic information, (2) blood tests including serological biomarkers (inflammatory, genetic and autoimmune), (3) physical exam, (4) patient questionnaires, (5) X-rays of hands and feet and (6) a musculoskeletal ultrasound scan.

Demographic data consisted of sex assigned at birth, age (in years), smoking exposure (ever), early morning stiffness duration (in minutes), Visual Analogue Scale (VAS) for fatigue (0–100), general health patient score (0–100) and global pain (0–100).

Blood tests consisted of anti-CCP antibodies (initially analysed using ImmunoCAP 250 (Phadia) with a positive cut-off of >7 IU/mL and later on, Bioplex 2200 CCP (BioRAD) cutoff >2.99 IU/mL. High titre was defined as ≥3 times the upper limit of normality), rheumatoid factor (RF) (initial cut-off for positivity ≥40 IU/mL and later ≥20 IU/mL), presence of anti-nuclear antibodies (ANA), presence of shared epitope (HLA DRB01*01, DRB01*04, DRB01*10 or a combination), C-reactive protein (CRP) concentration in mg/dL and erythrocyte sedimentation rate (ESR) (in mm/h).

The physical examination was performed by a rheumatologist and consisted of assessing the presence of joint tenderness to palpation. Although the presence of joint swelling was assessed, this was an exclusion criterion for joining the study, and hence individuals with clinical arthritis were not recruited. Joints assessed for tenderness were the following: temporomandibular joints, cervical spine, sternoclavicular joints, acromioclavicular joints, shoulders, elbows, wrists, metacarpo-phalangeal (MCP) joints 1–5, proximal interphalangeal (PIP) joints 1–5, hips, knees, ankles, talocalcaneal joints, midtarsal joints and metatarso-phalangeal (MTP) joints 1–5.

The patient questionnaires included a mannequin where participants indicated the joints where they had experienced pain during the previous week. This was referred to as ‘patient-reported joint pain’ (PRJP). The joints included were neck, back, shoulders, elbows, wrists, thumbs, hands/fingers (2-5), hips, knees, ankles and feet/toes. Participants also provided VAS scores (ranging from 0 to 100, with 100 being the most intense) regarding ‘global pain’, ‘general health’ and ‘abnormal fatigue’.

The joints scanned for the presence of PD, grey scale (GS) or erosions were shoulders, elbows, wrists, MCPs1-5, PIPs1-5, knees, ankles, talocalcaneal, midtarsal joints and MTP1-5. For wrists, examination included the radiocarpal, intercarpal and ulnocarpal joints. However, given that tenderness and PRJP referred to the wrist as a single anatomical region, ultrasound findings from these compartments were combined in order to simplify data interpretation. When at least one of these joints showed abnormalities, the highest grade of GS, PD or erosions was recorded to represent the wrist as a whole.

The tendons that were scanned as part of the study were the extensor carpi ulnaris and the flexor tendons of fingers 2–5 of both hands. These latter ones were assessed at both the MCP and PIP levels. Because tenosynovitis may affect only a segment of the tendon, these were recorded independently for analysis.

The study protocol suffered amendments and not all these sites were assessed in every participant: shoulders, talocalcaneal and midtarsal joints were removed from the protocol at early stages of the study, so they were not included in this analysis. Ultrasound scans of elbows, knees and ankles were not part of the initial protocol; they were added later on and therefore not assessed in the very first participants. Tendons were not initially part of the protocol either; they were added to the study ultrasound assessments later on.

Ultrasound scans were performed blindly by either a musculoskeletal ultrasonographer or a rheumatologist experienced in musculoskeletal ultrasound from a single centre. The three sonographers (KS, BS and LH) held a Postgraduate Certificate in musculoskeletal ultrasound and had a minimum of 1 year’s musculoskeletal ultrasound experience (often several years in general ultrasound). The rheumatologist performing the scans (RJW) had 23 years of experience, was European Federation of Societies for Ultrasound in Medicine and Biology level 3, British Society for Rheumatology and European Alliance of Associations for Rheumatology (EULAR) trainer. Before performing study scans, each ultrasound operator received training for the study protocol (a general research ultrasound guidance/atlas was used) and underwent a 4-week calibration period to standardise acquisition of images and ensure alignment of scoring system. When each operator joined the study, they were also assessed by a senior sonographer for scoring reliability. Although formal interobserver reliability exercises were not conducted specifically for the CCP study, in addition to this comprehensive protocol training, quarterly audits were performed whereby one patient scan per operator from the preceding quarter was jointly reviewed for image quality and scoring (ie, GS, PD, etc). Any discrepancies were discussed and used to reinforce scoring consistency and minimise operator variability.

The following machine models were used throughout the duration of the trial: (1) Philips (ATL HDI 5000) with 5–12 MHz and 8–15 MHz transducers, (2) General Electric S7 with a 6–15 MHz transducer, (3) General Electric Logiq E9 with a 6–15 MHz transducer. PD was set up with a pulse repetition frequency (PRF) of 700–1000 Hz, and a Doppler frequency of 6 MHz and 10 MHz for the Philips (ATL HDI 5000) and the two General Electric models, respectively.^4 7^ Sensitivity analyses were performed between the first two ultrasound machines.^4^

Ultrasound scans were assessed using a semiquantitative method initially proposed by EULAR, with scores ranging from 0 to 3 for GS and PD,^8^ and more recently following the EULAR/Outcome Measures in Rheumatology (OMERACT) scoring system.^9^ Assessment of the presence of bone erosions followed OMERACT definition.^10^ The size of bone erosions (defined as the diameter of the cortical break) was scored following a semi-quantitative scale (from 0 to 3).^11^ For the tendons, the presence of tenosynovitis in ultrasound was defined as OMERACT^10^ present/absent, and later on, following the later OMERACT consensus, a semi-quantitative scoring system (ranging from 0 to 3) was added for B-mode and for PD.^12^ Images were scored at the time of data acquisition.

### Statistical analysis

When participants attended their baseline appointment, ideally, they would have an ultrasound scan on the same day as the rest of the assessments. However, primarily due to logistical constraints, approximately a quarter of participants had this performed on a different date. This would result in some individuals having the scans separated by days/weeks from their baseline clinical appointment with the consequent protocol deviation. Considering that these are individuals at-risk (but not with established clinical arthritis), inflammation is expected to fluctuate. Hence, having different assessments days apart is likely to have a big impact on their association, which will be major or smaller depending on the patient. In order to avoid this bias, only participants who had all the assessments on the same day were included in the analysis. Additionally, in order to assess a potential selection bias, demographic, laboratory, clinical and ultrasound characteristics of the subjects that were and were not included in the analysis were compared.

In order to match the symptomatic joints with the ultrasound outcomes, only assessments of the following joints were analysed (table 1): for the physical exam: wrists, MCPs 1–5, PIPs 1–5, elbows, knees, ankles and MTPs 1–5. For the ultrasound scan: wrists, MCPs 1–5, PIPs 1–5, elbows, knees, ankles and MTPs 1–5. For PRJP: wrists, thumbs, hands/fingers, elbows, knees, ankles and feet/toes.

**Table 1:** Musculoskeletal assessments

	**Ultrasound of joints**	Physical exam	PRJP
	Elbow	Elbow	Elbow
Extensor carpi ulnaris	Wrist	Wrist	Wrist
	MCP1	MCP1	Thumb
	PIP1	PIP1	
MCP2 flexor tendon	MCP2	MCP2	Hand/fingers
MCP3 flexor tendon	MCP3	MCP3	
MCP4 flexor tendon	MCP4	MCP4	
MCP5 flexor tendon	MCP5	MCP5	
PIP2 flexor tendon	PIP2	PIP2	
PIP3 flexor tendon	PIP3	PIP3	
PIP4 flexor tendon	PIP4	PIP4	
PIP5 flexor tendon	PIP5	PIP5	
	Knee	Knee	Knee
	Ankle	Ankle	Ankle
	MTP1	MTP1	Foot/toes
	MTP2	MTP2	
	MTP3	MTP3	
	MTP4	MTP4	
	MTP5	MTP5	

MCP, metacarpo-phalangeal; MTP, metatarso-phalangeal; PIP, proximal interphalangeal; PRJP, Patient-reported joint pain.

In the physical exam and the patient questionnaire, the variables ‘tenderness’ and ‘painful’ respectively were dichotomous (yes/no). Ultrasound outcomes were also dichotomised to facilitate clinical interpretation. The variables GS, PD and erosions had four grades (0–3) and were coded as follows, based on the presence of inflammatory changes: GS ≤1 was coded as ‘no’ and GS >1 was coded as ‘yes’. PD=0 was coded as ‘no’ and PD ≥1 was coded as ‘yes’. Erosions=0 was coded as ‘no’ and erosions ≥1 was coded as ‘yes’. For analysis purposes, the variable ‘tenosynovitis’ was coded as present/absent following OMEARCT definition.^10^

Statistical analyses were performed using SPSS Statistics V.29.0.1.0 and R Studio V.2022.07.2+576. For descriptive statistics, the following variables were recorded as dichotomous based on literature review, expert opinion and international cutoffs: female (yes/no), smoking exposure (yes/no), shared epitope (positive/negative), high anti-CCP titre (yes/no), RF (positive/negative) and ANA (positive/negative).

Normality of continuous variables (ie, CRP, ESR, EMS, VAS scores) was assessed using Shapiro-Wilk test. For those with a normal distribution, mean and SD were calculated; otherwise, median and IQR. Comparisons of participants’ characteristics were performed using either Fisher’s exact test or χ² for dichotomised categorical variables, and independent t-test or Mann-Whitney U test for continuous variables as appropriate.

Although information about hand dominance was not recorded in the study, the majority of the population are right-handed and therefore symptoms and pathology secondary to mechanical reasons are more likely to be present on that side. In order to evidence any potential variations, the prevalence of symptoms/signs and ultrasound abnormalities were reported combined for right and left side joints, but also separately.

Joint-specific analyses were considered the primary outcomes of interest. Joint-level relationships between (a) ‘tenderness’, (b) ‘PRJP’ and (c) ‘both tenderness and PRJP’, and the presence of PD/GS/tenosynovitis/erosions were analysed using generalised estimating equations (GEE) logistic models. Joints were clustered by patient to account for correlation within the same person and were adjusted for age and sex as potential confounders, given their known associations with musculoskeletal symptoms and ultrasound abnormalities.^13 14^ Although additional covariates were considered, the number of events at the joint level was limited; therefore, inclusion of multiple variables in the models would have risked overfitting and unstable estimates. Therefore, model stability and interpretability were prioritised over extensive adjustment. Outcomes were binary for the presence/absence of PD, GS, tenosynovitis and erosions, as explained above. Population-averaged (pooled) GEE models were additionally used as secondary analyses to how joint tenderness and PRJP relate to ultrasound abnormalities across all joints. These analyses were cross-sectional and descriptive and no inferences regarding risk prediction or disease progression were intended.

To avoid selective modelling and inflation of type I error from multiple unadjusted tests, one joint-level model per ultrasound outcome was fitted and included joint location as a categorical factor, covering 19 joints (wrist; MCP1–5; PIP1–5; MTP1–5; elbow; knee; ankle). An exchangeable working correlation and robust (sandwich) standard errors were used. Analyses were complete-case at the outcome level, including all joints with observed data. Two-sided p values <0.05 were considered statistically significant for pooled effects.

The positive predictive value (PPV) and negative predictive value (NPV) of joint clinical information for tenderness and PRJP were calculated for each ultrasound abnormality. For each joint-outcome pair, right and left sides were pooled at the count level (summing true/false positives and true/false negatives) to derive a single PPV and NPV per joint. 95% CIs were calculated using Wilson methods.

This study analysed baseline data only, as the aim was to evaluate cross-sectional associations between symptoms/signs and concurrent ultrasound abnormalities. Longitudinal changes in ultrasound findings were beyond the scope of the present work, which focused only on assessments at the time of presentation.

## Results

A total of 451 participants were recruited for the ‘CCP Study’, and 323 had the three baseline joint assessments (physical exam, patient questionnaires and ultrasound scan) performed on the same day and were included in this analysis. At baseline, mean age was 50.2 years (SD 13.53); 70% (226/323) were female; 57.6% (186/323) had smoking exposure and 65.6% (196/299) were shared epitope positive. From a serological point of view, 42.4% (117/276) were RF positive; 61.3% (198/323) were anti-CCP high titre and 20.3% (48/237) were ANA positive. Median CRP was 4.0 mg/dL (IQR 2.2–7.3) and ESR 12.0 mm/hour (IQR 6.0–22.8). Symptom-wise, median early morning stiffness was 15.0 min (IQR 0–60.0), mean abnormal fatigue (VAS) score was 36.1 (SD 29.7), general health (VAS) score was 26.4 (SD22.6) and global pain (VAS) score was 31.4 (SD 25.5).

Missing data were as follows: shared epitope (7.4%); RF (14.5%); ANA (26.6%); CRP (9%); ESR (7.1%); early morning stiffness (12.3%); abnormal fatigue (6.5%); general health (5.9%); global pain (5.2%). Participants who underwent the ultrasound examination on a different date (128/451) did not differ significantly from those included in this analysis regarding demographic, laboratory, clinical characteristics and ultrasound findings (online supplemental table S1).

Regarding longitudinal information of the individuals analysed, 26% (84/323) of them developed RA, corresponding to an incidence rate of 5.73 per 100 person-years (95% CI 4.57 to 7.09). Among those who progressed, the median time to RA development was 53.5 weeks (IQR 24–165).

At the baseline appointment, ultrasound scans of elbows, knees and ankles were not performed in 64, 56 and 65 participants, respectively, as they were not part of the initial protocol; therefore, a total of 11 904 joints were analysed. Tendons were not initially part of the protocol either, and hence were only assessed in 153 participants.

**Figure 1:**
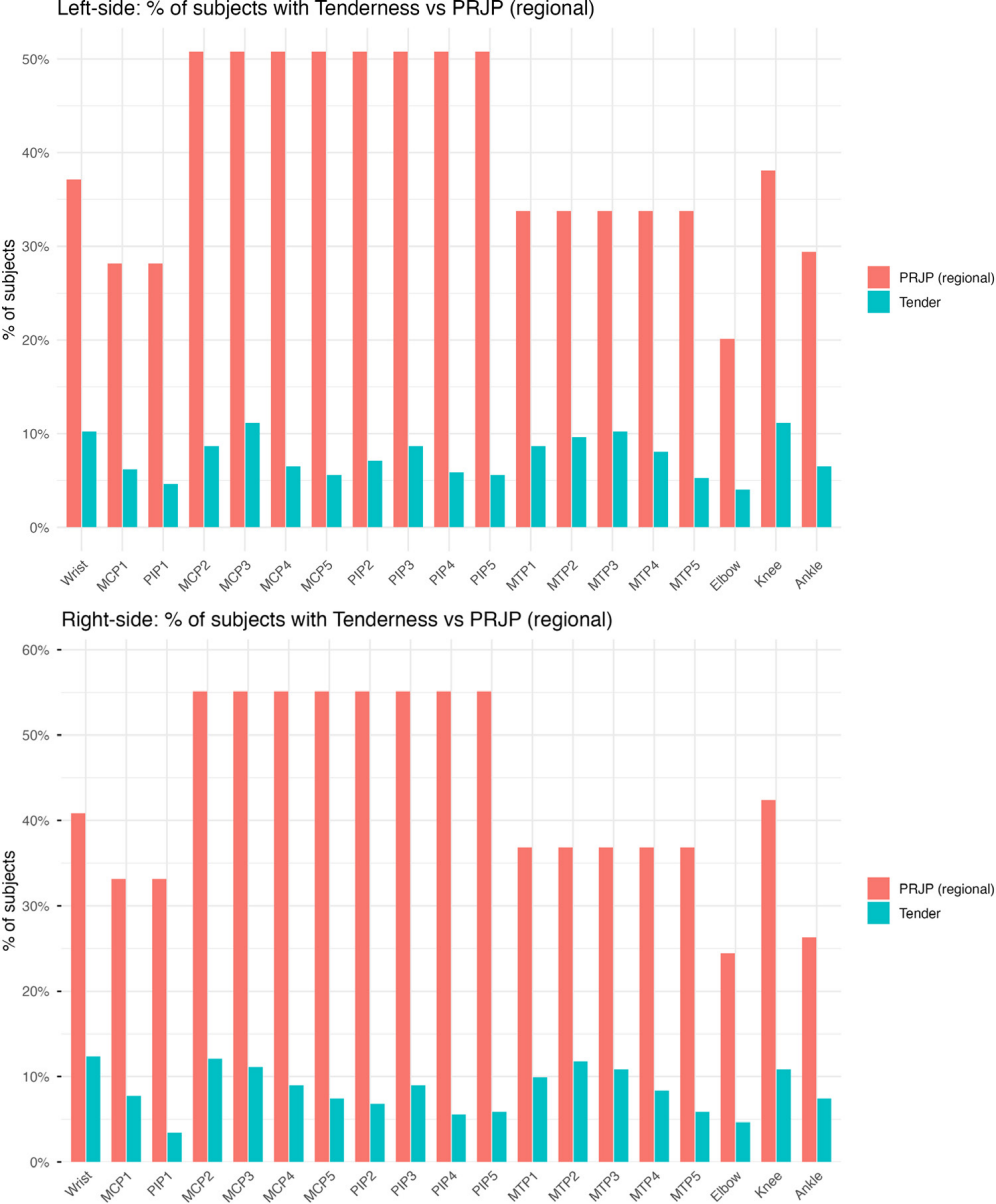
Prevalence of tenderness and PRJP per joint in percentage (left and right sides). MCP, metacarpo-phalangeal; MTP, metatarso-phalangeal; PIP, proximal interphalangeal; PRJP, patient-reported joint pain.

**Figure 2:**
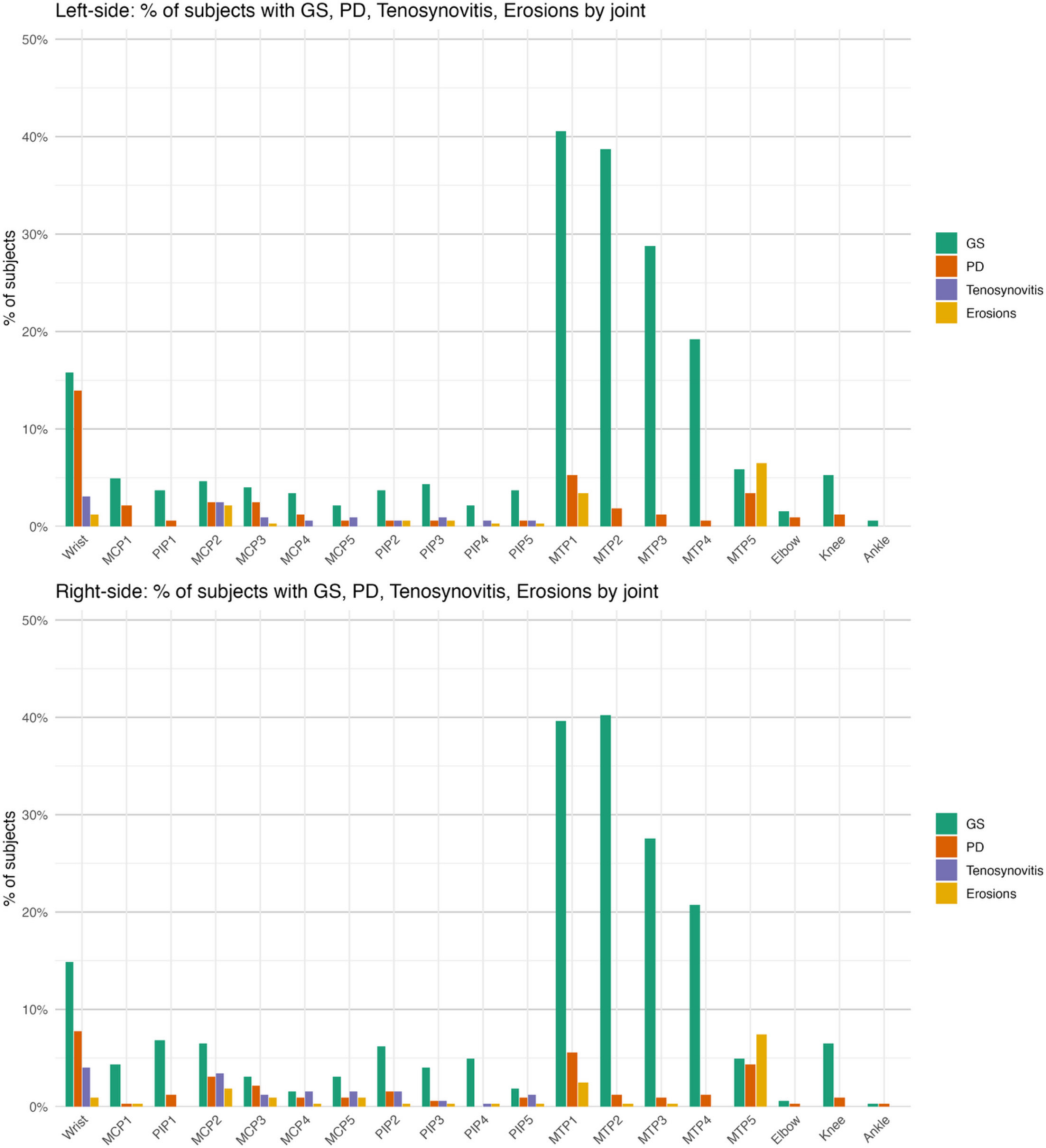
Prevalence of ultrasound abnormalities per joint in percentage (left and right sides). GS, grey scale; MCP, metacarpo-phalangeal; MTP, metatarso-phalangeal; PD, power Doppler; PIP, proximal interphalangeal.

The prevalence of symptoms/signs based on the location is shown in figure 1. The prevalence of ultrasound abnormalities based on the joint location is displayed in figure 2. Online supplemental table S2 and S3 show the prevalence of ultrasound abnormalities in each joint based on the presence of either tenderness, pain or both.

The prevalence of symptoms, signs and ultrasound abnormalities was slightly higher in the right side, likely due to mechanical factors. Unexpectedly, the prevalence of PD in the left wrist was higher than in the right one (14% vs 8%, respectively).

The pooled joint-level GEE models (adjusted for sex and age) showed a strong association between tenderness and ultrasound abnormalities, with joint tenderness being significantly associated with PD, GS, erosions and tenosynovitis.

On the contrary, PRJP showed a weaker association with ultrasound findings in general, and especially for erosions; whose association was not statistically significant. Joint estimates are displayed in table 2.

**Table 2:** Pooled joint-level associations between joint tenderness/PRJP and ultrasound abnormalities

Variable	Ultrasound abnormality	OR (95% CI)	P value
Tenderness	GS	4.45 (3.72 to 5.32)	<0.001***
	PD	5.55 (3.74 to 8.26)	<0.001***
	Tenosynovitis	2.31 (1.25 to 4.30)	0.008**
	Erosions	4.86 (3.14 to 7.52)	<0.001***
PRJP	GS	1.50 (1.09 to 2.08)	0.013*
	PD	2.31 (1.10 to 4.88)	0.027*
	Tenosynovitis	2.11 (1.04 to 4.30)	0.040*
	Erosions	1.93 (0.94 to 2.96)	0.073

Estimates are OR (95% CI) p value from pooled joint-level GEE logistic models, adjusted for age and sex and accounting for clustering of joints within participants.

* p≤0.05; **p≤0.01; ***p≤0.001.

GEE, generalised estimating equations; GS, grey scale; PD, power Doppler; PRJP, patient-reported joint pain.

At the individual joint level, adjusted joint-specific GEE logistic models showed consistent association between tenderness and the presence of ultrasound abnormalities (table 3): wrist (p=0.001), PIP2 (p=0.050), PIP3 (p=0.024), PIP5 (p<0.001), elbow (p=0.001) and MTP3 (p=0.004) tenderness were associated with GS changes. This association was even more significant for PD; showing that joint tenderness was associated with inflammatory changes in wrist (p<0.001), MCP4 (p=0.016), MCP5 (p=0.005), PIP2 (p<0.001), PIP5 (p<0.001), elbow (p=0.012), knee (p=0.016), MTP1 (p<0.001), MTP2 (p<0.001) and MTP3 (p=0.002). The association between tenderness and tenosynovitis was limited, showing statistical significance only in the wrist (p=0.024). As far as erosions are concerned, they were associated with tenderness at the levels of PIP2 (p=0.039), PIP3 (p<0.001), PIP5 (p<0.001) and MTP1 (p=0.006), suggesting that local tenderness on examination corresponds to structural damage on ultrasound, particularly in the small joints.

**Table 3:** Prediction of ultrasound abnormalities based on the location of tenderness

Location	GS	PD	Tenosynovitis	Erosions
Wrist	3.16 (1.64 to 6.10) 0.001***	4.03 (1.98 to 8.21) <0.001***	3.70 (1.19 to 11.52) 0.024*	3.59 (0.59 to 21.88) 0.166
MCP1	2.47 (1.64 to 6.10) 0.127	5.28 (0.74 to 37.66) 0.097	N/A	--
MCP2	1.80 (0.60 to 5.33) 0.292	1.94 (0.50 to 7.49) 0.339	1.96 (0.51 to 7.51) 0.326	2.60 (0.51 to 13.20) 0.249
MCP3	2.02 (0.60 to 6.74) 0.254	2.52 (0.62 to 10.30) 0.198	4.93 (0.91 to 26.68) 0.064	3.48 (0.39 to 31.06) 0.265
MCP4	0.90 (0.11 to 7.18) 0.922	9.52 (1.51 to 60.02) 0.016*	--	--
MCP5	0.94 (0.12 to 7.52) 0.956	10.61 (2.04 to 55.14) 0.005**	--	--
PIP1	1.00 (0.00 to 255.63) 1.00	1.00 (0.00 to 255.63) 1.00	N/A	--
PIP2	3.42 (1.00 to 11.74) 0.050*	38.55 (5.85 to 254.06) <0.001***	--	5.74 (1.09 to 30.30) 0.039*
PIP3	3.34 (1.18 to 9.48) 0.024*	4.98 (0.43 to 57.99) 0.200	--	176.55 (11.02 to 2827.55) <0.001***
PIP4	0.83 (0.11 to 6.06) 0.858	--	17.04 (0.64 to 451.18) 0.090	--
PIP5	8.49 (2.68 to 26.87) <0.001***	26.49 (4.22 to 166.31) <0.001***	--	21.02 (6.09 to 72.48) <0.001***
Elbow	24.68 (3.52 to 172.97) 0.001***	39.78 (2.24 to 705.69) 0.012*	N/A	--
Knee	2.46 (0.96 to 6.30) 0.061	7.48 (1.45 to 38.45) 0.016*	N/A	--
Ankle	10.25 (0.83 to 127.07) 0.070	--	N/A	--
MTP1	1.70 (0.88 to 3.28) 0.113	5.47 (2.23 to 13.42) <0.001***	N/A	5.57 (1.63 to 19.04) 0.006**
MTP2	1.71 (0.99 to 2.94) 0.054	9.95 (2.95 to 33.58) <0.001***	N/A	--
MTP3	2.23 (1.30 to 3.84) 0.004**	7.64 (2.17 to 26.83) 0.002**	N/A	--
MTP4	1.03 (0.45 to 2.37) 0.946	5.33 (0.96 to 29.67) 0.056	N/A	--
MTP5	2.53 (0.68 to 9.47) 0.168	3.36 (0.89 to 12.73) 0.074	N/A	3.06 (0.95 to 9.81) 0.060

Estimates are OR (95% CI) p value from joint-specific GEE logistic models, adjusted for age and sex and accounting for clustering within participants.

‘--’ not estimable.

* p≤0.05; **p≤0.01; ***p≤0.001.

GEE, generalised estimating equations; GS, grey scale; MCP, metacarpo-phalangeal; MTP, metatarso-phalangeal; N/A, not applicable; PD, power Doppler; PIP, proximal interphalangeal.

In contrast, although PRJP also showed association with ultrasound findings, this was a more selective pattern in adjusted joint-specific GEE logistic models (table 4). For GS, significant association with PRJP was present in MCP5 (p=0.008), PIP2 (p=0.022), PIP5 (p=0.003), elbow (p=0.008), MTP1 (p=0.015), MTP2 (p=0.030), MTP3 (p=0.014) and MTP4 (p=0.046). PRJP showed association with PD in the wrist (p=0.004), PIP2 (p=0.037), elbow (p=0.023), MTP1 (p=0.001) and MTP2 (p=0.033). No significant associations were evidenced for PRJP and tenosynovitis and association with erosions was only at the MTP5 joint (p=0.005).

**Table 4:** Prediction of ultrasound abnormalities based on the location of PRJP

Location		GS	PD	Tenosynovitis	Erosions
Wrist	Wrist	1.40 (0.86 to 2.26) 0.175	2.30 (1.30 to 4.07) 0.004**	1.52 (0.58 to 3.98) 0.399	1.57 (0.32 to 7.68) 0.574
Hand	MCP1	1.34 (0.61 to 2.93) 0.470	2.80 (0.66 to 11.94) 0.165	N/A	--
	MCP2	1.60 (0.78 to 3.29) 0.198	1.89 (0.67 to 5.32) 0.230	1.93 (0.75 to 4.96) 0.172	1.21 (0.40 to 3.66) 0.740
	MCP3	2.04 (0.79 to 5.25) 0.141	1.12 (0.36 to 3.46) 0.850	2.15 (0.36 to 12.77) 0.398	3.16 (0.15 to 65.48) 0.458
	MCP4	1.63 (0.59 to 4.51) 0.343	2.56 (0.61 to 10.79) 0.199	5.37 (0.58 to 49.91) 0.140	--
	MCP5	15.95 (2.00 to 111.60) 0.008**	3.68 (0.42 to 32.04) 0.238	2.61 (0.54 to 12.51) 0.231	14.45 (0.55 to 382.94) 0.110
	PIP1	0.87 (0.40 to 1.93) 0.739	4.44 (0.66 to 29.87) 0.125	N/A	--
	PIP2	2.73 (1.15 to 6.46) 0.022*	6.59 (1.12 to 38.83) 0.037*	5.33 (0.60 to 47.54) 0.134	--
	PIP3	1.95 (0.70 to 5.40) 0.200	1.07 (0.15 to 7.56) 0.945	3.75 (0.32 to 43.54) 0.291	--
	PIP4	2.11 (0.74 to 6.02) 0.162	--	--	--
	PIP5	20.21 (2.79 to 146.48) 0.003**	--	5.20 (0.51 to 53.32) 0.165	--
Elbow	Elbow	10.19 (1.84 to 56.46) 0.008**	12.75 (1.42 to 114.33) 0.023*	N/A	--
Knee	Knee	1.06 (0.48 to 2.33) 0.881	2.97 (0.52 to 16.95) 0.221	N/A	--
Ankle	Ankle	5.74 (0.55 to 59.49) 0.143	--	N/A	--
Foot	MTP1	1.64 (1.10 to 2.44) 0.015*	4.51 (1.92 to 10.61) 0.001***	N/A	1.49 (0.52 to 4.25) 0.459
	MTP2	1.55 (1.04 to 2.29) 0.030*	4.82 (1.14 to 20.42) 0.033*	N/A	--
	MTP3	1.07 (1.12 to 2.59) 0.014*	3.47 (0.60 to 19.96) 0.163	N/A	--
	MTP4	1.63 (1.01 to 2.65) 0.046*	8.57 (0.93 to 79.33) 0.058	N/A	--
	MTP5	1.48 (0.72 to 3.03) 0.283	1.65 (0.74 to 3.68) 0.224	N/A	2.74 (1.36 to 5.52) 0.005**

Estimates are OR (95% CI) p value from joint-specific GEE logistic models, adjusted for age and sex and accounting for clustering within participants.

‘--’ not estimable.

* p≤0.05; **p≤0.01; ***p≤0.001.

GEE, generalised estimating equations; GS, grey scale; MCP, metacarpo-phalangeal; MTP, metatarso-phalangeal; N/A, not applicable; PD, power Doppler; PIP, proximal interphalangeal; PRJP, patient-reported joint pain.

Simultaneous presence of tenderness and PRJP in one joint generally was not significantly more associated with ultrasound abnormalities at the joint level compared with tenderness alone (online supplemental table S4).

Pooling right and left sides, tenderness and PRJP had limited PPVs for ultrasound abnormalities (table 5 and online supplemental table S5, respectively), with tenderness slightly outperforming PRJP.

**Table 5:** Predictive values for the presence of ultrasound abnormalities based on the location of joint tenderness (pooled side for right and left sides)

Location of joint tenderness		Joint GS	Joint PD	Tenosynovitis	Erosions
Wrist	PPV	30.1	24.7	17.5	2.7
	NPV	86.6	90.9	94	99.1
MCP1	PPV	8.9	4.4	N/A	2.2
	NPV	95.7	99	N/A	100
MCP2	PPV	9	4.5	10.3	3
	NPV	94.8	97.4	94.2	98.1
MCP3	PPV	5.6	4.2	6.1	1.4
	NPV	96.7	97.9	98.2	99.5
MCP4	PPV	2	4	0	0
	NPV	97.5	99.2	97.5	99.8
MCP5	PPV	2.4	4.8	0	0
	NPV	97.4	99.5	97.2	99.5
PIP1	PPV	0	0	N/A	0
	NPV	94.5	99	N/A	100
PIP2	PPV	11.1	8.9	0	6.7
	NPV	95.5	99.5	97.6	100
PIP3	PPV	8.8	1.8	0	3.5
	NPV	96.3	99.5	98.2	99.8
PIP4	PPV	2.7	0	5.3	0
	NPV	96.4	100	99.3	99.7
PIP5	PPV	13.5	8.1	0	2.7
	NPV	97.9	99.7	97.9	99.8
MTP1	PPV	12.5	8.3	N/A	0
	NPV	99.2	99.6	N/A	100
MTP2	PPV	12.5	4.7	N/A	0
	NPV	93.6	99.1	N/A	100
MTP3	PPV	3.2	3.2	N/A	0
	NPV	99.6	100	N/A	100
MTP4	PPV	51.7	18.3	N/A	10
	NPV	61	95.9	N/A	97.8
MTP5	PPV	50.7	7.2	N/A	0
	NPV	61.7	99.1	N/A	99.8
Elbow	PPV	44.1	4.4	N/A	0
	NPV	73.6	99.3	N/A	99.8
Knee	PPV	18.9	3.8	N/A	0
	NPV	79.9	99.3	N/A	100
Ankle	PPV	11.1	11.1	N/A	16.7
	NPV	94.9	96.5	N/A	93.6

Data are in percentage.

GS, grey scale; MCP, metacarpo-phalangeal; MTP, metatarso-phalangeal; N/A, not applicable; NPV, negative predictive value; PD, power Doppler; PIP, proximal interphalangeal; PPV, positive predictive value.

For GS changes, the predictive values of symptoms and signs were variable, with MTPs presenting the highest PPV and also the lowest NPVs, suggesting that GS in the feet is harder to rule out clinically.

In contrast, absence of individual signs (tenderness) (table 5) or individual symptoms (PRJP) (online supplemental table S5) had consistently high NPVs for PD, tenosynovitis and erosions across all joints. This was particularly higher for tenderness, with values >93% for any of these abnormalities and >96% for PD, with the exception of the wrist (91%).

## Discussion

Association between physical examination and ultrasound findings in individuals with established RA has been thoroughly investigated. However, this is the first study to assess whether the presence of local joint symptoms/signs correspond to the presence of ultrasound abnormalities at the joint level in individuals at-risk of RA who do not have clinical arthritis. The purpose of the study was not to predict progression to an inflammatory arthritis but to describe cross-sectional associations between local signs/symptoms and concomitant ultrasound abnormalities in this at-risk population. These findings should be interpreted as exploratory and hypothesis-generating, and require validation in independent prospective cohorts before any implications for practice can be considered.

Using joint-level population-averaged GEE models, joint tenderness showed a strong association with ultrasound abnormalities (PD, GS, tenosynovitis and erosions); in contrast, the association of PRJP with ultrasound findings was weaker and less consistent.

When focusing on individual joints, overall, tenderness demonstrated a stronger and more widespread correlation with ultrasound abnormalities compared with PRJP. Associations with PD, which usually reflects active synovial inflammation, were particularly prominent in the small joints, which are typical sites involved in RA (particularly in early disease).^13^ In contrast, PRJP association with ultrasound abnormalities appeared to be more inconsistent, making it a less reliable marker of local subclinical inflammation. Furthermore, the NPV of lack of tenderness for PD, tenosynovitis and erosions was high for all joints, implying that in the absence of tenderness, the presence of these abnormalities is unlikely. The significant association between tenderness and ultrasound abnormalities, specifically inflammatory ones, combined with its high NPV may justify future prospective studies assessing whether clinically guided scanning approaches may be feasible in this at-risk population.

Whereas pooled GEE analysis showed a strong association between tenderness and ultrasound abnormalities at the joint-level, estimates for individual joints were often not statistically significant. This is likely due to the limited number of events and reduced statistical power in specific anatomical sites, rather than lack of association. Some joint-specific estimates were imprecise, with wide CIs reflecting low event counts.

In patients with RA, when focusing on the ‘joint level’, most studies agree that there is an acceptable association between joint swelling and the presence of PD and/or GS on ultrasound, at least in certain locations.^15^,^18^ However, the relationship between joint tenderness and ultrasound abnormalities is more controversial: a cross-sectional study in RA focused on the wrist and reported a moderate association between tenderness and ultrasound changes in patients who had arthritis in the joint.^19^ However, another study reported that this association was not present in the absence of joint swelling.^20^ Whereas other RA studies did not find a significant association between tenderness and ultrasound synovitis,^16 17^ this discrepancy with the current study could be due to the difference in the population investigated (‘RA’ vs ‘at-risk’ in the current one). This contention is supported by a study that assessed the association between the presence of PD and joint tenderness in non-swollen joints in RA^21^: they found this to be significant in patients with disease duration <2 years but not in those with >5 years of disease duration, perhaps due to postinflammatory pain or even an element of fibromyalgia, both present in long-standing disease.

Similarly to ultrasound, subclinical inflammation on MRI also appears to be associated with tenderness at the joint level.^22^ This was reported by a study including participants fulfilling the EULAR definition of ‘arthralgia suspicious for progression to RA’^23^; however, a sub-analysis showed that for anti-CCP positive individuals, it was bone marrow oedema and not synovitis or tenosynovitis that was independently associated with tenderness. Likewise, we did not find a consistent association between tenosynovitis and tenderness. When looking at the specific characteristics of their population, their median number of tender joints and VAS scores for pain were considerably higher than in our study (likely because patients with a high level of VAS, that is, fibromyalgia were not included in ours), which could explain the discrepancies regarding the association between synovitis and tenderness.

PRJP alone or in combination with tenderness added little to the optimisation of symptom-guided ultrasound scans. Whereas PRJP was associated with ultrasound changes in the feet, other studies have found a low and modest association between PRJP and ultrasound abnormalities in RA patients depending on the joint assessed.^16 17 19^ It has been suggested that patient outcome measures such as patient-reported global pain or factors like disability or low mental health can have an influence on PRJP or even on tenderness.^24 25^ In order to assess the impact of this, additional analyses were performed, including the patient global pain VAS score as a confounding factor. However, it did not have a significant effect on the association of ultrasound abnormalities (online supplS7). A possible explanation is that individuals with fibromyalgia, who usually have disproportionate VAS scores or a high perception of pain, were not included in the present study. Also, at-risk individuals have little experience with joint pain, whereas RA patients will be more familiar with arthralgia. This could result in different thresholds for pain and better recognition of joint symptoms. Most importantly, the low prevalence of PD at baseline suggests that PRJP is often the reflection of non-specific musculoskeletal symptoms in this at-risk group and not a surrogate of subclinical disease, which has also been suggested by other studies.^22^

PRJP in at-risk individuals is more prevalent than tenderness (figure 1). Therefore, using lack of tenderness over lack of PRJP facilitates the identification of more joints that may not benefit from an ultrasound scan. This reduction of scans was estimated to be between 88% and 95%, depending on the joint, when compared with the systematic performance of hands and feet. These findings suggest that routine ultrasound scans of non-tender joints (eg, hands and feet) may have a limited yield, although this would require prospective validation. The only questionable exception to this would be wrists, whose involvement is a hallmark of RA and PD was present in around 5%–10% of subjects with no symptoms in the right and left wrist, respectively, decreasing the NPV of lack of symptoms or signs to 90% in that location. As previous research has shown, the extent of subclinical synovitis with PD correlates with the number of risk factors^3^ and neither PRJP, tenderness nor ultrasound abnormalities are specific for inflammatory arthritis and may be influenced by mechanical or degenerative factors.

Our findings complement prior work in at-risk cohorts. Rogier *et al* reported that a negative ultrasound scan performed only in tender joints had a high NPV for the development of an inflammatory arthritis.^26^ Consistently, in our cohort, the absence of tenderness or PRJP yielded very high NPVs for ultrasound negativity, suggesting that clinical assessment and ultrasound identify overlapping low-risk subgroups.

Previous work from our research group reported the predictive value of bone erosions on ultrasound, particularly at the MTP5 for the development of an inflammatory arthritis.^7^ Complementary to this, the current study has shown that erosions in this location can be associated with PRJP.

Bone erosions are traditionally considered to be the endpoint of chronic synovial inflammation; therefore, their presence in individuals at-risk of RA without clinical disease may appear paradoxical. However, a number of mechanisms can explain this: first, subclinical synovitis is a common finding in at-risk individuals and can precede clinical arthritis.^4^ Additionally, this can fluctuate or resolve over time,^3^ implying that structural changes may persist despite lack of inflammation at a specific time point. Second, synovitis induces the production of pro-inflammatory cytokines, which together with auto-antibodies can stimulate osteoclast differentiation, potentially leading to focal bone damage even in the absence of clinical disease.^27 28^ These immune-mediated bone changes have also been evidenced on MRI,^22^ where bone marrow oedema can occur prior to clinical synovitis, and predicts structural progression.^29^ Finally, non-inflammatory factors, such as mechanical stress (which is particularly relevant in weight-bearing joints such as the MTP joints) may also contribute to cortical irregularities or degenerative changes. These can mimic erosions and take place simultaneously in at-risk individuals.^11^

This study has a number of methodological limitations. First, it was a retrospective cross-sectional analysis of prospectively collected data; therefore, temporal relationships, causality and prognostic implications cannot be established. Second, although models were adjusted for age and sex, residual confounding cannot be excluded; as well as other potential confounders that were not included in the joint-level models. Third, in the PRJP questionnaires, participants could not always indicate each individual joint but rather a group of joints affected (ie, hand pain instead of specific MCP or PIP joints). This reduced precision in mapping PRJP to ultrasound sites may partly explain the weaker association observed. Fourth, ultrasound scores were dichotomised to facilitate interpretability and reflect decision-making in real-world settings; however, this may have reduced sensitivity to differences in the inflammatory burden. Fifth, protocol amendments during the course of the study resulted in variations in some joints and tendons assessed across participants, which may have influenced comparisons between anatomical sites. Sixth, the long recruitment period and use of different ultrasound machines may have introduced variability in image acquisition, particularly as Doppler sensitivity has improved over time. Finally, formal intraobserver and interobserver/rater reliability testing was not performed, which may have introduced variability despite standardised training procedures. Although this is an inherent limitation of multiclinician studies conducted over long periods of time, this heterogeneity may also increase the generalisability of the findings to routine clinical practice.

Ultrasound assessment of individuals at-risk of RA is not yet routine practice, and its precise role in this setting has not yet been defined. However, anti-CCP positive individuals with non-specific musculoskeletal symptoms but no clinical arthritis are frequently found in early arthritis pathways, and the uncertainty about the presence of subclinical inflammation is a common challenge. Therefore, understanding how local symptoms and signs relate to concomitant ultrasound abnormalities may still be clinically informative.

Within this context, our findings suggest that joint tenderness, (and to a lesser degree PRJP) is associated with ultrasound abnormalities at the same anatomical site. These associations were not uniform across all joints or for all ultrasound features, evidencing the heterogeneity of symptoms-imaging relationships in at-risk populations.

The consistently high NPVs observed across most joints suggest that in absence of local joint symptoms or signs, the presence of ultrasound abnormalities was uncommon; however, these results should be interpreted cautiously as they may be influenced by the low prevalence of some of these abnormalities.

It is important to highlight that these findings do not constitute recommendations for ultrasound scanning strategies or clinical decision-making; instead, they provide data that may help inform future prospective studies evaluating whether joint symptom/sign-guided ultrasound scans could improve feasibility and efficiency of ultrasound assessments in at-risk populations. Future research should also investigate whether integrating other biomarkers into predictive models may contribute to composite risk-stratification tools, ultimately improving early diagnosis and prevention strategies in at-risk populations.

## Conclusions

Joint tenderness was associated with local inflammatory changes on ultrasound, predominantly in the small joints; however, the association of PRJP with ultrasound abnormalities was more limited. Additionally, in the absence of joint tenderness, the presence of PD, tenosynovitis or bone erosions on ultrasound were uncommon. These results may inform future studies evaluating symptom/sign-guided ultrasound assessment approaches in at-risk individuals.

## Supplementary material

10.1136/bmjopen-2026-117366 online supplemental file 1

## Data Availability

Data are available on reasonable request.

## References

[1] Mankia K, Di Matteo A, Emery P (2020). Prevention and cure: The major unmet needs in the management of rheumatoid arthritis. J Autoimmun.

[2] Di Matteo A, Duquenne L, Cipolletta E (2022). Ultrasound subclinical synovitis in anti-CCP-positive at-risk individuals with musculoskeletal symptoms: an important and predictable stage in the rheumatoid arthritis continuum. Rheumatology (Sunnyvale).

[3] Garcia-Montoya L, Kang J, Duquenne L (2024). Factors associated with resolution of ultrasound subclinical synovitis in anti-CCP-positive individuals with musculoskeletal symptoms: a UK prospective cohort study. Lancet Rheumatol.

[4] Nam JL, Hensor EMA, Hunt L (2016). Ultrasound findings predict progression to inflammatory arthritis in anti-CCP antibody-positive patients without clinical synovitis. Ann Rheum Dis.

[5] Naredo E, Valor L, De la Torre I (2013). Ultrasound Joint Inflammation in Rheumatoid Arthritis in Clinical Remission: How Many and Which Joints Should Be Assessed?. *Arthritis Care & Research*.

[6] Di Matteo A, De Lorenzis E, Duquenne L (2024). Ultrasound in anti-CCP+ at-risk individuals without clinical synovitis: development of a novel 6-joint protocol for feasible risk prediction. Rheumatology (Oxford).

[7] Di Matteo A, Mankia K, Duquenne L (2020). Ultrasound erosions in the feet best predict progression to inflammatory arthritis in anti-CCP positive at-risk individuals without clinical synovitis. Ann Rheum Dis.

[8] Maria Antonietta D’Agostino MA, Wakefield RJ, Filipucci E (2005). Intra- and inter-observer reliability of ultrasonography for detecting and scoring synovitis in rheumatoid arthritis: a report of a EULAR ECSISIT task force. Ann Rheum Dis.

[9] D’Agostino M-A, Terslev L, Aegerter P (2017). Scoring ultrasound synovitis in rheumatoid arthritis: a EULAR-OMERACT ultrasound taskforce**-**Part 1: definition and development of a standardised, consensus-based scoring system. RMD Open.

[10] Wakefield RJ, Balint PV, Szkudlarek M (2005). Musculoskeletal ultrasound including definitions for ultrasonographic pathology. J Rheumatol.

[11] Wakefield RJ, Gibbon WW, Conaghan PG (2000). The value of sonography in the detection of bone erosions in patients with rheumatoid arthritis: a comparison with conventional radiography. Arthritis Rheum.

[12] Naredo E, D’Agostino MA, Wakefield RJ (2013). Reliability of a consensus-based ultrasound score for tenosynovitis in rheumatoid arthritis. Ann Rheum Dis.

[13] Khidir SJH, van Dijk BT, Krijbolder DI (2023). Joint involvement in RA starts predominantly in the hands: functional, clinical and imaging studies in clinically suspect arthralgia and during progression to RA. RMD Open.

[14] Myasoedova E, Crowson CS, Kremers HM (2010). Is the incidence of rheumatoid arthritis rising?: Results from Olmsted County, Minnesota, 1955–2007. Arthritis & Rheumatism.

[15] Janta I, Naredo E, Martinez-Estupinan L (2013). Patient self-assessment and physician’s assessment of rheumatoid arthritis activity: which is more realistic in remission status? A comparison with ultrasonography. Rheumatology (Sunnyvale).

[16] Hammer HB, Michelsen B, Sexton J (2019). Swollen, but not tender joints, are independently associated with ultrasound synovitis: results from a longitudinal observational study of patients with established rheumatoid arthritis. Ann Rheum Dis.

[17] Hirata A, Ogura T, Hayashi N (2017). Concordance of Patient‐Reported Joint Symptoms, Physician‐Examined Arthritic Signs, and Ultrasound‐Detected Synovitis in Rheumatoid Arthritis. *Arthritis Care & Research*.

[18] Quintana-López G, Maldonado-Cañón K, Flórez-Suárez JB (2021). Correlation and agreement between physical and ultrasound examination after a training session dedicated to the standardization of synovitis assessment in rheumatoid arthritis patients. *Adv Rheumatol*.

[19] Baan H, Hoekstra M, Veehof M (2011). Ultrasound findings in rheumatoid wrist arthritis highly correlate with function. Disabil Rehabil.

[20] Tan YK, Moorakonda RB, Allen JC (2019). Back to the basics: Understanding joint swelling and tenderness at the wrist in rheumatoid arthritis through the use of ultrasonography. Int J Rheum Dis.

[21] Gessl I, Popescu M, Schimpl V (2021). Role of joint damage, malalignment and inflammation in articular tenderness in rheumatoid arthritis, psoriatic arthritis and osteoarthritis. Ann Rheum Dis.

[22] Burgers LE, ten Brinck RM, van der Helm-van Mil AHM (2019). Is joint pain in patients with arthralgia suspicious for progression to rheumatoid arthritis explained by subclinical inflammation? A cross-sectional MRI study. Rheumatology (Sunnyvale).

[23] Ruta S, Prado ES, Chichande JT (2020). EULAR definition of “arthralgia suspicious for progression to rheumatoid arthritis” in a large cohort of patients included in a program for rapid diagnosis: role of auto-antibodies and ultrasound. Clin Rheumatol.

[24] Cheung PP, Gossec L, Ruyssen-Witrand A (2013). The relationship of patient-reported joints with active synovitis detected by power Doppler ultrasonography in rheumatoid arthritis. Clin Exp Rheumatol.

[25] Felbo SK, Wiell C, Østergaard M (2022). Do tender joints in active psoriatic arthritis reflect inflammation assessed by ultrasound and magnetic resonance imaging?. *Rheumatology (Oxford*).

[26] Rogier C, Frazzei G, Kortekaas MC (2022). An ultrasound negative for subclinical synovitis in arthralgia patients: is it helpful in identifying those not developing arthritis?. *Rheumatology (Oxford*).

[27] Schett G, Gravallese E (2012). Bone erosion in rheumatoid arthritis: mechanisms, diagnosis and treatment. Nat Rev Rheumatol.

[28] Harre U, Georgess D, Bang H (2012). Induction of osteoclastogenesis and bone loss by human autoantibodies against citrullinated vimentin. J Clin Invest.

[29] McQueen FM, Ostendorf B (2006). What is MRI bone oedema in rheumatoid arthritis and why does it matter. *Arthritis Res Ther*.

