# Supplementary material for: Association between joint tenderness, patient-reported joint pain and ultrasound abnormalities in anti-CCP positive individuals at risk of rheumatoid arthritis: a cross-sectional study from a Leeds (UK) cohort
Source: BMJ Open. 2026 Jun 4;16(6):e117366. doi: 10.1136/bmjopen-2026-117366 (PMC13239518; doi:10.1136/bmjopen-2026-117366)
Supplement: online supplemental file 1 [file bmjopen-16-6-s001.docx]

SUPPLEMENTARY

MATERIAL

**Supplementary table S1**. Baseline characteristics of the participants recruited for the CCP study, split according to inclusion in the analysis.

|  | **PARTICIPANTS INCLUDED**  **(n=323)** | **PARTICIPANTS**  **NOT**  **INCLUDED**  **(n=128)** | **P value** |
| --- | --- | --- | --- |
| Age, years | 50.2 (13.53) | 50.1 (11.90) | 0.957 |
| Female | 226 (70.0%) | 96 (75.0%) | 0.429 |
| Smoking exposure | 186 (57.6%) | 74 (57.8%) | 1.000 |
| SE positive | 196 (65.6%) | 73 (60.8%) | 0.365 |
| High Anti-CCP titre | 198 (61.3%) | 67 (52.3%) | 0.062 |
| RF positive | 117 (42.4%) | 41 (40.2%) | 0.679 |
| ANA positive | 48 (20.3%) | 14 (15.6%) | 0.428 |
| CRP, mg/dL | 4.0 (2.2-7.3) | 4.0 (0.2-4.0) | 0.088 |
| ESR, mm/h | 12.0 (6.0-22.8) | 10.0 (4.0-20.0) | 0.497 |
| EMS, minutes | 15.0 (0-60.0) | 5.0 (0-30.0) | 0.127 |
| Abnormal fatigue (VAS) score | 36.1 (29.7) | 35.5 (32.4) | 0.856 |
| General health (VAS) score | 26.4 (22.6) | 23.2 (29.8) | 0.274 |
| Global pain (VAS) score | 31.4 (25.5) | 27.3 (34.1) | 0.212 |
| Participants with ≥1 tender joint | 178 (55.1%) | 67 (52.3%) | 0.594 |
| Participants with ≥1 painful region | 307 (95%) | 120 (93.7%) | 0.440 |
| Participants with ≥1 joint with GS | 246 (76.3%) | 107 (83.6%) | 0.085 |
| Participants with ≥1 joint with PD | 96 (29.7%) | 37 (28.9%) | 0.874 |
| Participants with ≥1 joint site with TS | 38 (24.8%) | 17 (23.6%) | 0.734 |
| Participants with ≥1 bone erosion | 54 (16.7%) | 14 (10.9%) | 0.119 |

Data are n (%), mean (SD) or median (IQR).

ANA= antinuclear antibodies; Anti-CCP= anti-cyclic citrullinated peptide; CRP= C-reactive protein; EMS= early morning stiffness; ESR= erythrocyte sedimentation rate; GS= grey scale; IQR= interquartile range; PD= Power Doppler; PRJP= patient-reported joint pain; TS=tenosynovitis; SE= standard deviation; SE= shared epitope; VAS= visual analogue scale.

Missing data for subjects included in the analysis: SE (7.4%); RF (14.5%); ANA (26.6%); CRP (9%); ESR (7.1%); EMS (12.3%); Abnormal fatigue (6.5%); General health (5.9%); Global pain (5.2%).

Missing data for subjects excluded from the analysis: SE (6.3%); RF (20.3%); ANA (29.7%); CRP (7.9%); ESR (5.6%); EMS (5.6%); Abnormal fatigue (6.9%); General health (5.4%); Global pain (5.6%).

**Supplementary table S2.** Prevalence of ultrasound abnormalities based on the location and the presence of joint tenderness, pain (PRJP) or both. – Right side

| Joint | Symptom/  sign | Presence of joint PD | Presence of joint GS | Presence of tenosynovitis | Erosions |
| --- | --- | --- | --- | --- | --- |
| Wrist | Tenderness | 17.5 (7/40) | 30.0 (12/40) | 13.6 (3/22) | 2.5 (1/40) |
|  | Pain | 10.6 (14/132) | 15.9 (21/132) | 8.2 (5/61) | 1.5 (2/132) |
|  | Both T+P | 20.0 (6/30) | 36.7 (11/30) | 7.1 (1/14) | 3.3 (1/30) |
| MCP1 | Tenderness | 0 (0/25) | 8.0 (2/25) | N/A | 4.0 (1/25) |
|  | Pain | 0 (0/107) | 5.6 (6/107) | N/A | 0 (0/107) |
|  | Both T+P | 0 (0/20) | 5.0 (1/20) | N/A | 0 (0/20) |
| MCP2 | Tenderness | 0 (0/39) | 2.6 (1/39) | 15.8 (3/19) | 2.6 (1/39) |
|  | Pain | 3.4 (6/177) | 6.7 (12/178) | 9.6 (8/83) | 1.7 (3/178) |
|  | Both T+P | 0 (0/38) | 2.6 (1/38) | 16.7 (3/18) | 2.6 (1/38) |
| MCP3 | Tenderness | 2.8 (1/36) | 0 (0/36) | 6.3 (1/16) | 2.8 (1/36) |
|  | Pain | 2.2 (4/178) | 2.8 (5/178) | 2.4 (2/83) | 1.1 (2/178) |
|  | Both T+P | 3.1 (1/32) | 0 (032) | 6.7 (1/15) | 3.1 (1/32) |
| MCP4 | Tenderness | 3.4 (1/29) | 0 (0/29) | 0 (0/14) | 0 (0/29) |
|  | Pain | 1.7 (3/178) | 1.7 (3/178) | 4.8 (4/83) | 0.6 (1/178) |
|  | Both T+P | 3.8 (1/26) | 0 (0/26) | 0 (0/11) | 0 (0/26) |
| MCP5 | Tenderness | 8.3 (2/24) | 4.2 (1/24) | 0 (0/10) | 0 (0/24) |
|  | Pain | 1.7 (3/178) | 5.6 (10/178) | 4.8 (4/83) | 1.1 (2/178) |
|  | Both T+P | 9.5 (2/21) | 4.8 (1/21) | 0 (0/9) | 0 (0/21) |
| PIP1 | Tenderness | 0 (0/11) | 0 (0/11) | N/A | 0 (0/11) |
|  | Pain | 3.7 (4/107) | 6.5 (7/107) | N/A | 0 (0/107) |
|  | Both T+P | 0 (0/7) | 0 (0/7) | N/A | 0 (0/7) |
| PIP2 | Tenderness | 13.6 (3/22) | 18.2 (4/22) | 0 (0/8) | 4.5 (1/22) |
|  | Pain | 2.8 (5/178) | 8.4 (15/178) | 6.0 (5/83) | 0.6 (1/178) |
|  | Both T+P | 14.3 (3/21) | 19.0 (4/21) | 0 (0/8) | 4.8 (1/21) |
| PIP3 | Tenderness | 3.4 (1/29) | 3.4 (1/29) | 0 (0/14) | 0 (0/29) |
|  | Pain | 1.1 (2/178) | 4.5 (8/178) | 1.2 (1/83) | 0.6 (1/178) |
|  | Both T+P | 3.8 (1/26) | 3.8 (1/26) | 0 (0/11) | 0 (0/26) |
| PIP4 | Tenderness | 0 (0/18) | 5.6 (1/18) | 0 (0/8) | 0 (0/18) |
|  | Pain | 0 (0/178) | 6.7 (12/178) | 1.2 (1/83) | 0.6 (1/178) |
|  | Both T+P | 0 (0/15) | 6.7 (1/15) | 0 (0/6) | 0 (0/15) |
| PIP5 | Tenderness | 10.5 (2/19) | 10.5 (2/19) | 0 (0/8) | 5.3 (1/19) |
|  | Pain | 1.7 (3/178) | 2.8 (5/178) | 4.8 (4/83) | 0.6 (1/178) |
|  | Both T+P | 12.5 (2/16) | 12.5 (2/16) | 0 (0/6) | 6.3 (1/16) |

| Joint | Symptom/  sign | Presence of joint PD | Presence of joint GS | Presence of tenosynovitis | Erosions |
| --- | --- | --- | --- | --- | --- |
| MTP1 | Tenderness | 18.8 (6/32) | 46.9 (15/32) | N/A | 9.4 (3/32) |
|  | Pain | 11.8 (11/119) | 48.7 (58/119) | N/A | 2.5 (3/119) |
|  | Both T+P | 25.0 (6/24) | 50.0 (12/24) | N/A | 8.3 (2/24) |
| MTP2 | Tenderness | 7.9 (3/38) | 50.0 (19/38) | N/A | 0 (0/38) |
|  | Pain | 2.5 (3/119) | 42.9 (51/119) | N/A | 0 (0/119) |
|  | Both T+P | 11.1 (3/27) | 44.4 (12/27) | N/A | 0 (0/27) |
| MTP3 | Tenderness | 8.6 (3/35) | 48.6 (17/35) | N/A | 0 (0/35) |
|  | Pain | 2.5 (3/119) | 31.1 (37/119) | N/A | 0.8 (1/119) |
|  | Both T+P | 12.5 (3/24) | 50.0 (12/24) | N/A | 0 (0/24) |
| MTP4 | Tenderness | 3.7 (1/27) | 18.5 (5/27) | N/A | 0 (0/27) |
|  | Pain | 3.4 (4/119) | 24.4 (29/119) | N/A | 0 (0/119) |
|  | Both T+P | 4.8 (1/21) | 23.8 (5/21) | N/A | 0 (0/21) |
| MTP5 | Tenderness | 5.3 (1/19) | 5.3 (1/19) | N/A | 15.8 (3/19) |
|  | Pain | 5.9 (7/119) | 5.0 (6/119) | N/A | 12.6 (15/119) |
|  | Both T+P | 6.3 (1/16) | 6.3 (1/16) | N/A | 12.5 (2/16) |
| Elbow | Tenderness | 0 (0/13) | 7.7 (1/13) | N/A | 0 (0/13) |
|  | Pain | 1.6 (1/64) | 3.1 (2/64) | N/A | 0 (0/64) |
|  | Both T+P | 0 (0/9) | 11.1 (1/9) | N/A | 0 (0/9) |
| Knee | Tenderness | 0 (0/31) | 12.9 (4/31) | N/A | 0 (0/31) |
|  | Pain | 1.7 (2/117) | 7.7 (9/117) | N/A | 0 (0/117) |
|  | Both T+P | 0 (0/30) | 10.0 (3/30) | N/A | 0 (0/30) |
| Ankle | Tenderness | 5.9 (1/17) | 5.9 (1/17) | N/A | 0 (0/17) |
|  | Pain | 1.4 (1/71) | 1.4 (1/71) | N/A | 0 (0/71) |
|  | Both T+P | 7.7 (1/13) | 7.7 (1/13) | N/A | 0 (0/13) |

Data are % (n/N)

GS= grey scale; MCP= metacarpo-phalangeal; MTP= metatarso-phalangeal; N/A= not applicable; PD= power Doppler; PIP= proximal interphalangeal; T+P= simultaneous presence of joint tenderness and pain.

**Supplementary table S3.** Prevalence of ultrasound abnormalities based on the location and the presence of joint tenderness, pain (PRJP) or both. – Left side

| Joint | Symptom | Presence of joint PD | Presence of joint GS | Presence of tenosynovitis | Erosions |
| --- | --- | --- | --- | --- | --- |
| Wrist | Tenderness | 33.3 (11/33) | 30.3 (10/33) | 22.2 (4/18) | 3.0 (1/33) |
|  | Pain | 19.2 (23/120) | 18.3 (22/120) | 9.1 (5/55) | 0.8 (1/120) |
|  | Both T+P | 33.3 (8/24) | 29.2 (7/24) | 15.4 (2/13) | 4.2 (1/24) |
| MCP1 | Tenderness | 10.0 (2/20) | 10.0 (2/20) | N/A | 0 (0/20) |
|  | Pain | 4.4 (4/91) | 5.5 (5/91) | N/A | 0 (0/91) |
|  | Both T+P | 14.3 (2/14) | 14.3 (2/14) | N/A | 0 (0/14) |
| MCP2 | Tenderness | 10.7 (3/28) | 17.9 (5/28) | 0 (0/10) | 3.6 (1/28) |
|  | Pain | 3.6 (6/164) | 6.7 (11/164) | 6.1 (5/82) | 2.4 (4/164) |
|  | Both T+P | 8.7 (2/23) | 17.4 (4/23) | 0 (0/10) | 4.3 (1/23) |
| MCP3 | Tenderness | 5.6 (2/36) | 11.1 (4/36) | 5.9 (1/17) | 0 (0/36) |
|  | Pain | 2.4 (4/164) | 6.7 (11/164) | 3.7 (3/82) | 0.6 (1/164) |
|  | Both T+P | 6.3 (2/32) | 12.5 (4/32) | 6.7 (1/15) | 0 (0/32) |
| MCP4 | Tenderness | 4.8 (1/21) | 4.8 (1/21) | 0 (0/11) | 0 (0/21) |
|  | Pain | 1.2 (2/164) | 4.3 (7/164) | 2.4 (2/82) | 0 (0/164) |
|  | Both T+P | 6.3 (1/16) | 6.3 (1/16) | 0 (0/8) | 0 (0/16) |
| MCP5 | Tenderness | 0 (0/18) | 0 (0/18) | 0 (0/11) | 0 (0/18) |
|  | Pain | 0.6 (1/164) | 3.7 (6/164) | 2.4 (2/82) | 0 (0/164) |
|  | Both T+P | 0 (0/12) | 0 (0/12) | 0 (0/7) | 0 (0/12) |
| PIP1 | Tenderness | 0 (0/15) | 0 (0/15) | N/A | 0 (0/15) |
|  | Pain | 0 (0/91) | 3.3 (3/91) | N/A | 0 (0/91) |
|  | Both T+P | 0 (0/10) | 0 (0/10) | N/A | 0 (0/10) |
| PIP2 | Tenderness | 4.3 (1/23) | 4.3 (1/23) | 0 (0/11) | 8.7 (2/23) |
|  | Pain | 0.6 (1/164) | 5.5 (9/164) | 1.2 (1/82) | 1.2 (2/164) |
|  | Both T+P | 4.8 (1/21) | 4.8 (1/21) | 0 (0/11) | 9.5 (2/21) |
| PIP3 | Tenderness | 0 (0/28) | 14.3 (4/28) | 0 (0/14) | 7.1 (2/28) |
|  | Pain | 0 (0/164) | 6.1 (10/164) | 3.7 (3/82) | 1.2 (2/164) |
|  | Both T+P | 0 (0/25) | 16.0 (4/25) | 0 (0/13) | 8.0 (2/25) |
| PIP4 | Tenderness | 0 (0/19) | 0 (0/19) | 9.1 (1/11) | 0 (0/19) |
|  | Pain | 0 (0/164) | 2.4 (4/164) | 2.4 (2/82) | 0.6 (1/164) |
|  | Both T+P | 0 (0/18) | 0 (0/18) | 10.0 (1/10) | 0 (0/18) |
| PIP5 | Tenderness | 5.6 (1/18) | 16.7 (3/18) | 0 (0/10) | 0 (0/18) |
|  | Pain | 1.2 (2/164) | 7.3 (12/164) | 1.2 (1/82) | 0.6 (1/164) |
|  | Both T+P | 5.9 (1/17) | 17.6 (3/17) | 0 (0/9) | 0 (0/17) |

| Joint | Symptom | Presence of joint PD | Presence of joint GS | Presence of tenosynovitis | Erosions |
| --- | --- | --- | --- | --- | --- |
| MTP1 | Tenderness | 17.9 (5/28) | 57.1 (16/28) | N/A | 10.7 (3/28) |
|  | Pain | 9.2 (10/109) | 44.0 (48/109) | N/A | 4.6 (5/109) |
|  | Both T+P | 17.4 (4/23) | 52.2 (12/23) | N/A | 8.7 (2/23) |
| MTP2 | Tenderness | 6.5 (2/31) | 51.6 (16/31) | N/A | 0 (0/31) |
|  | Pain | 3.7 (4/109) | 45.9 (50/109) | N/A | 0 (0/109) |
|  | Both T+P | 8.3 (2/24) | 58.3 (14/24) | N/A | 0 (0/24) |
| MTP3 | Tenderness | 0 (0/33) | 39.4 (13/33) | N/A | 0 (0/33) |
|  | Pain | 0.9 (1/109) | 35.8 (39/109) | N/A | 0 (0/109) |
|  | Both T+P | 0 (0/26) | 46.2 (12/26) | N/A | 0 (0/26) |
| MTP4 | Tenderness | 3.8 (1/26) | 19.2 (5/26) | N/A | 0 (0/26) |
|  | Pain | 0.9 (1/109) | 22.0 (24/109) | N/A | 0 (0/109) |
|  | Both T+P | 5.6 (1/18) | 27.8 (5/18) | N/A | 0 (0/18) |
| MTP5 | Tenderness | 17.6 (3/17) | 17.6 (3/17) | N/A | 17.6 (3/17) |
|  | Pain | 4.6 (5/109) | 8.3 (9/109) | N/A | 10.1 (11/109) |
|  | Both T+P | 13.3 (2/15) | 13.3 (2/15) | N/A | 13.3 (0/15) |
| Elbow | Tenderness | 18.2 (2/11) | 18.2 (2/11) | N/A | 0 (0/11) |
|  | Pain | 3.6 (2/55) | 5.5 (3/55) | N/A | 0 (0/55) |
|  | Both T+P | 12.5 (1/8) | 12.5 (1/8) | N/A | 0 (0/8) |
| Knee | Tenderness | 9.1 (3/33) | 12.1 (4/33) | N/A | 0 (0/33) |
|  | Pain | 1.9 (2/106) | 5.7 (6/106) | N/A | 0 (0/106) |
|  | Both T+P | 6.5 (2/31) | 9.7 (3/31) | N/A | 0 (0/31) |
| Ankle | Tenderness | 0 (0/14) | 0 (0/14) | N/A | 0 (0/14) |
|  | Pain | 0 (0/78) | 1.3 (1/78) | N/A | 0 (0/78) |
|  | Both T+P | 0 (0/13) | 0 (0/13) | N/A | 0 (0/13) |

Data are % (n/N)

GS= grey scale; MCP= metacarpo-phalangeal; MTP= metatarso-phalangeal; N/A= not applicable; PD= power Doppler; PIP= proximal interphalangeal; T+P= simultaneous presence of joint tenderness and pain.

**Supplementary table S4.** Prediction of ultrasound abnormalities based on the location of the simultaneous presence of tenderness and PRJP.

| **LOCATION** | **GS** | **PD** | **TENOSYNOVITIS** | **EROSIONS** |
| --- | --- | --- | --- | --- |
| Wrist | 2.79 (1.39-5.6) 0.004** | 3.58 (1.72-7.44) 0.001*** | 1.20 (0.29-4.88) 0.801 | 6.14 (1.03-36.78) 0.047* |
| MCP1 | 2.16 (0.54-8.62) 0.276 | 5.03 (0.40-63.51) 0.212 | N/A | -- |
| MCP2 | 1.25 (0.32-4.91) 0.748 | 1.20 (0.22-6.48) 0.829 | 2.69 (0.54-13.35) 0.226 | 2.88 (0.67-12.28) 0.153 |
| MCP3 | 2.07 (0.49-8.84) 0.326 | 2.43 (0.48-12.42) 0.286 | 5.69 (1.07-30.33) 0.042* | 4.54 (0.57-36.31) 0.154 |
| MCP4 | 1.06 (0.16-6.85) 0.951 | 13.03 (2.06-82.26) 0.006** | -- | -- |
| MCP5 | 1.17 (0.06-23.94) 0.919 | 16.06 (2.58-99.83) 0.003** | -- | -- |
| PIP1 | -- | -- | N/A | -- |
| PIP2 | 1.72 (0.19-15.80) 0.632 | 24.13 (4.65-125.19) <0.001*** | -- | -- |
| PIP3 | 4.04 (1.53-10.68) 0.005** | 5.94 (0.57-61.88) 0.136 | -- | 21.59 (0.44-1057.44) 0.122 |
| PIP4 | 0.20 (0.00-126.79) 0.624 | -- | 30.24 (0.84-1083.14) 0.062 | -- |
| PIP5 | 7.85 (2.05-29.97) 0.003** | 29.79 (4.80-184.84) <0.001*** | -- | -- |
| Elbow | 15.87 (1.89-133.61) 0.011* | 11.81 (1.52-91.85) 0.018** | N/A | -- |
| Knee | 2.00 (0.80-5.00) 0.137 | 3.85 (0.64-23.02) 0.139 | N/A | -- |
| Ankle | -- | -- | N/A | -- |
| MTP1 | 1.83 (0.91-3.66) 0.088 | 5.86 (2.38-14.43) <0.001*** | N/A | 5.65 (1.61-19.80) 0.007** |
| MTP2 | 1.81 (1.02-3.19) 0.041* | 16.35 (4.17-64.06) <0.001*** | N/A | -- |
| MTP3 | 2.82 (1.63-4.88) <0.001*** | 11.43 (2.85-45.82) 0.001*** | N/A | -- |
| MTP4 | 1.71 (0.87-3.36) 0.110 | 7.37 (1.22-44.54) 0.030* | N/A | -- |
| MTP5 | 1.99 (0.40-10.01) 0.402 | 2.13 (0.33-13.96) 0.429 | N/A | 2.30 (0.64-8.26) 0.201 |

Estimates are OR (95%CI) p-value from joint-specific GEE logistic models adjusted for age and sex, accounting for clustering of joints within participants. *p≤0.05; ** p≤0.01; ***p ≤0.001.

CI = Confidence interval; GEE= Generalised Estimating Equations GS= grey scale; MCP= metacarpo-phalangeal; MTP= metatarso-phalangeal; N/A= not applicable; OR= odds ratio; PD= power Doppler; PIP= proximal interphalangeal; PRJP= patient-reported joint pain; “--” not estimable.

**Supplementary table S5.** Predictive values for the presence of US abnormalities based on the location of joint PRJP (pooled side for right and left sides)

| Location of PRJP | | Joint GS | Joint PD | Tenosynovitis | Erosions |
| --- | --- | --- | --- | --- | --- |
| Wrist | PPV | 17.1 | 14.7 | 8.6 | 1.2 |
|  | NPV | 85.8 | 91.6 | 93.2 | 99 |
| MCP1 | PPV | 5.6 | 2 | N/A | 0 |
|  | NPV | 95.8 | 99.1 | N/A | 99.8 |
| MCP2 | PPV | 6.7 | 3.5 | 7.9 | 2 |
|  | NPV | 95.7 | 98 | 95.7 | 98 |
| MCP3 | PPV | 4.7 | 2.3 | 3 | 0.9 |
|  | NPV | 97.7 | 97.7 | 98.6 | 99.7 |
| MCP4 | PPV | 2.9 | 1.5 | 3.6 | 0.3 |
|  | NPV | 98 | 99.3 | 99.3 | 100 |
| MCP5 | PPV | 4.7 | 1.2 | 3.6 | 0.6 |
|  | NPV | 99.7 | 99.7 | 98.6 | 99.7 |
| PIP1 | PPV | 5.1 | 2 | N/A | 0 |
|  | NPV | 94.6 | 99.6 | N/A | 100 |
| PIP2 | PPV | 7 | 1.8 | 3.6 | 0.9 |
|  | NPV | 97.4 | 99.7 | 99.3 | 100 |
| PIP3 | PPV | 5.3 | 0.6 | 2.4 | 0.9 |
|  | NPV | 97 | 99.3 | 99.3 | 100 |
| PIP4 | PPV | 4.7 | 0 | 1.8 | 0.6 |
|  | NPV | 97.7 | 100 | 100 | 100 |
| PIP5 | PPV | 5 | 1.5 | 3 | 0.6 |
|  | NPV | 99.7 | 100 | 99.3 | 100 |
| MTP1 | PPV | 4.2 | 2.5 | N/A | 0 |
|  | NPV | 99.5 | 99.7 | N/A | 100 |
| MTP2 | PPV | 6.7 | 1.8 | N/A | 0 |
|  | NPV | 92.6 | 99 | N/A | 100 |
| MTP3 | PPV | 1.3 | 0.7 | N/A | 0 |
|  | NPV | 99.7 | 100 | N/A | 100 |
| MTP4 | PPV | 46.5 | 10.5 | N/A | 3.5 |
|  | NPV | 63.2 | 97.4 | N/A | 97.4 |
| MTP5 | PPV | 44.3 | 3.1 | N/A | 0 |
|  | NPV | 63 | 99.3 | N/A | 99.8 |
| Elbow | PPV | 33.3 | 1.8 | N/A | 0.4 |
|  | NPV | 74.5 | 99.3 | N/A | 100 |
| Knee | PPV | 23.2 | 2.2 | N/A | 0 |
|  | NPV | 81.7 | 99.8 | N/A | 100 |
| Ankle | PPV | 6.6 | 5.3 | N/A | 11.4 |
|  | NPV | 95.2 | 96.9 | N/A | 95.4 |

Data are in %.

GS= grey scale; MCP= metacarpo-phalangeal; MTP= metatarso-phalangeal; N/A= not applicable; NPV= negative predictive value; PD= power doppler; PIP= proximal interphalangeal; PPV= positive predictive value; PRJP= patient-reported joint pain; US= ultrasound.

**Supplementary table S6.** Prediction of ultrasound abnormalities based on the location of tenderness, adjusted for global pain VAS scores.

| **LOCATION** | **GS** | **PD** | **TENOSYNOVITIS** | **EROSIONS** |
| --- | --- | --- | --- | --- |
| Wrist | 2.4 (1.16-4.94) 0.018* | 3.31 (1.56-7.01) 0.002** | 2.26 (0.74-6.90) 0.151 | 4.79 (0.74-30.86) 0.100 |
| MCP1 | 2.27 (0.67-7.73) 0.190 | 11.51 (2.6-51.08) 0.001** | N/A | -- |
| MCP2 | 1.10 (0.31-3.90) 0.883 | 1.62 (0.23-11.36) 0.625 | 3.35 (0.61-18.56) 0.166 | 3.17 (0.60-16.64) 0.173 |
| MCP3 | 1.42 (0.30-6.64) 0.657 | 2.90 (0.59-14.22) 0.189 | 7.47 (1.79-31.11) 0.006** | 9.36 (1.06-82.25) 0.044* |
| MCP4 | 0.86 (0.11-6.75) 0.885 | 7.28 (0.74-71.42) 0.089 | -- | -- |
| MCP5 | 1.10 (0.06-20.55) 0.951 | 11.05 (1.95-62.55) 0.007** | -- | -- |
| PIP1 | -- | -- | N/A | -- |
| PIP2 | 1.59 (0.21-12.29) 0.655 | 69.25 (8.3-577.59) <0.001*** | -- | -- |
| PIP3 | 4.24 (1.34-13.35) 0.014* | 11.74 (0.47-293.89) 0.134 | -- | 14.34 (0.21-996.94) 0.219 |
| PIP4 | -- | -- | 207.51 (0.68-631.67) 0.067 | -- |
| PIP5 | 7.64 (1.85-31.48) 0.005** | 24.25 (3.52-167.05) 0.001** | -- | 0.61 (0.00-105.83) 0.851 |
| Elbow | 20.26 (3.37-121.76) 0.001** | -- | N/A | -- |
| Knee | 2.67 (0.98-7.26) 0.054 | 6.36 (1.71-23.65) 0.006** | N/A | -- |
| Ankle | 13.71 (1.74-107.76) 0.013* | -- | N/A | -- |
| MTP1 | 1.43 (0.77-2.68) 0.261 | 4.39 (1.93-9.98) <0.001*** | N/A | 4.26 (1.38-13.17) 0.012* |
| MTP2 | 1.57 (0.89-2.77) 0.118 | 13.99 (4.26-45.91) <0.001*** | N/A | -- |
| MTP3 | 2.61 (1.55-4.38) <0.001*** | 7.79 (2.16-28.05) 0.002** | N/A | -- |
| MTP4 | 1.14 (0.56-2.33) 0.722 | 7.43 (1.09-50.47) 0.040* | N/A | -- |
| MTP5 | 2.52 (0.64-9.98) 0.187 | 2.89 (0.61-13.65) 0.180 | N/A | 3.26 (1.05-10.1) 0.041* |

Estimates are OR (95% CI) p-value from joint-specific GEE logistic models adjusted for age, sex and global pain VAS scores, accounting for clustering within participants. *p≤0.05; ** p≤0.01; ***p ≤0.001.

CI= confidence interval; GEE= Generalised Estimating Equations; GS= grey scale; MCP= metacarpo-phalangeal; MTP= metatarso-phalangeal; N/A= not applicable; OR= odds ratio; PD= power Doppler; PIP= proximal interphalangeal; VAS= visual analogue scale; “--” not estimable.

**Supplementary table S7.** Prediction of ultrasound abnormalities based on the location of PRJP, adjusted for global pain VAS scores.

| **LOCATION** | | **GS** | **PD** | **TENOSYNOVITIS** | **EROSIONS** |
| --- | --- | --- | --- | --- | --- |
| Wrist | Wrist | 1.30 (0.83-2.03) 0.259 | 1.99 (1.09-3.65) 0.025* | 1.23 (0.47-3.27) 0.671 | 1.98 (0.40-9.73) 0.401 |
| Hand | MCP1 | 1.09 (0.47-2.55) 0.838 | 2.26 (0.53-9.69) 0.273 | N/A | -- |
|  | MCP2 | 1.36 (0.65-2.82) 0.411 | 2.05 (0.69-6.06) 0.196 | 3.55 (1.03-12.29) 0.045* | 0.89 (0.21-3.88) 0.881 |
|  | MCP3 | 1.35 (0.53-3.43) 0.535 | 0.93 (0.36-2.38) 0.881 | 2.34 (0.40-13.56) 0.343 | 2.26 (0.09-59.70) 0.625 |
|  | MCP4 | 1.27 (0.36-4.52) 0.707 | 3.33 (0.30-36.46) 0.325 | 4.49 (0.51-39.26) 0.174 | -- |
|  | MCP5 | 13.69 (1.57-119.16) 0.018* | 3.68 (0.46-29.71) 0.222 | 1.34 (0.09-21.09) 0.833 | 14.27 (2.88-70.66) 0.001** |
|  | PIP1 | 1.15 (0.49-2.73) 0.746 | 3.34 (0.54-20.79) 0.196 | N/A | -- |
|  | PIP2 | 2.57 (1.14-5.82) 0.024* | -- | 54.85 (0.00-436.93) 0.206 | -- |
|  | PIP3 | 2.42 (0.89-6.54) 0.082 | 2.25 (0.21-23.76) 0.501 | 3.16 (0.21-46.91) 0.403 | -- |
|  | PIP4 | 2.64 (1.03-6.78) 0.044* | -- | -- | -- |
|  | PIP5 | 19.85 (3.02-130.33) 0.002** | -- | 6.30 (0.82-48.30) 0.076 | -- |
| Elbow | Elbow | 8.9 (1.6-49.58) 0.013* | 9.85 (1.28-75.92) 0.028* | N/A | -- |
| Knee | Knee | 1.17 (0.47-2.92) 0.734 | 2.26 (0.30-16.82) 0.428 | N/A | -- |
| Ankle | Ankle | 6.86 (0.77-61.26) 0.085 | -- | N/A | -- |
| Foot | MTP1 | 1.30 (0.90-1.89-1.62) 0.162 | 4.17 (1.82-9.57) <0.001*** | N/A | 1.36 (0.63-2.91) 0.430 |
|  | MTP2 | 1.37 (0.93-2.01) 0.109 | 3.96 (0.94-16.58) 0.060 | N/A | -- |
|  | MTP3 | 1.48 (0.97-2.25) 0.066 | 3.55 (0.62-20.15) 0.153 | N/A | -- |
|  | MTP4 | 1.39 (0.87-2.23) 0.17 | 8.01 (0.77-83.04) 0.081 | N/A | -- |
|  | MTP5 | 1.64 (0.82-3.31) 0.164 | 1.75 (0.82-3.73) 0.149 | N/A | 2.79 (1.35-5.74) 0.005** |

Estimates are OR (95% CI) p-value from joint-specific GEE logistic models adjusted for age, sex and global pain VAS scores, accounting for clustering within participants. *p≤0.05; ** p≤0.01; ***p ≤0.001.

CI= confidence interval; GEE= Generalised Estimating Equations; GS= grey scale; MCP= metacarpo-phalangeal; MTP= metatarso-phalangeal; N/A= not applicable; OR= odds ratio; PD= power Doppler; PIP= proximal interphalangeal; PRJP= patient-reported joint pain; VAS= visual analogue scale; “--” not estimable.
