# Supplementary material for: Association between joint tenderness, patient-reported joint pain and ultrasound abnormalities in anti-CCP positive individuals at risk of rheumatoid arthritis: a cross-sectional study from a Leeds (UK) cohort
Source: BMJ Open. 2026 Jun 4;16(6):e117366. doi: 10.1136/bmjopen-2026-117366 (PMC13239518; doi:10.1136/bmjopen-2026-117366)
Supplement: Supplementary file 2 [file bmjopen-2026-117366.reviewer_comments.pdf]

PEER REVIEW HISTORY

BMJ Open publishes all reviews undertaken for accepted manuscripts. Reviewers are asked to complete a checklist review form and are provided with free text boxes to elaborate on their assessment. These free text comments are reproduced below.

This paper was submitted to another journal from BMJ but declined for publication following peer review. The authors addressed the reviewers' comments and submitted the revised paper to BMJ Open. The paper was subsequently accepted for publication at BMJ Open.

ARTICLE DETAILS

Title (Provisional)

"Association between joint tenderness, patient-reported joint pain and ultrasound abnormalities in anti-CCP positive individuals at-risk of rheumatoid arthritis: a cross-sectional study from a Leeds (UK) cohort."

Authors

Garcia-Montoya, Leticia; Kang, Jing; Duquenne, Laurence; Di Matteo, Andrea; Harnden, Kate; Nam, Jacqueline Leong; Chowdhury, Rahaymin; Wakefield, Richard J; Mankia, Kulveer Singh; Emery, Paul

---

VERSION 1 - REVIEW

**Reviewer**	**1**
**Name**	**Reviewer 1**
**Affiliation**	
**Date**	**06-Jan-2026**
**COI**	**No competing interest**

Comments not published pending reviewer's consent

**Reviewer**	**2**
**Name**	**Reviewer 2**
**Affiliation**	
**Date**	**06-Jan-2026**
**COI**	**No competing interest**

Comments not published pending reviewer's consent

**Reviewer**	**3**
**Name**	**Reviewer 3**
**Affiliation**	
**Date**	**06-Jan-2026**
**COI**	**No competing interest**

Comments not published pending reviewer’s consent

VERSION 2 - REVIEW

**Reviewer**	**4**
**Name**	**Dack, Kyle**
**Affiliation**	**University of Bristol, MRC Integrative Epidemiology Unit**
**Date**	**06-Apr-2026**
**COI**	**None**

Statistical review summary:

The paper is well written and the methods/results reporting largely aligns with STROBE guidance. The statistical analyses are all reasonable and implemented well.

However, I have two concerns. First, the results omit some estimates based on statistical significance - this is discouraged because it risks introducing selective reporting bias. Second, there is a slight misalignment between the introduction and methods where the study is described as exploratory and descriptive, and the discussion where there is clear inference and recommendations for clinical practice. The interpretation therefore seems overly strong or confident, especially because minimizing confounding bias was not described as a part of the methods and appears limited to age and sex.

Abstract:

1. There are a few acronyms presented in the abstract before being defined, such as US and anti-CCP. Generally there are quite a lot of acronyms in the abstract which impair readability somewhat, and if any are non-essential (e.g. used infrequently, not standard clinical acronyms) it may be beneficial to reduce them if possible.

2. The setting is not reported, and I suggest adding this to the start of the methods.

Introduction:

3. The introduction is clear and summarises the rationale and context for the study nicely. My only suggestion relevant to the introduction (and rest of the paper) relates to that in Comment 1: consider if there are any non-essential acronyms. For example:

-US - I am unsure if this is a common, standard acronym for ultrasound, but the space saved is minimal, and at a cost of readability.

-EFSUMB, BME, FT - these and others are only used once in the paper.

-There is evidence that increased acronym use can impair readability, or increase risk of misinterpretation, so it would be beneficial to apply it to the most frequently used phrases where there will be space saved. https://pmc.ncbi.nlm.nih.gov/articles/PMC7556863/, https://pmc.ncbi.nlm.nih.gov/articles/PMC7556863/

Methods:

4. The data source is described (CCP), is it possible to also comment on the location of the study - is it entirely based in Leeds?

5. The description of the variables and statistical analyses is very clear, and decisions are explained/justified well. Regarding the models - it is not clear whether confounding bias was addressed. Models were adjusted for age and sex - was this to minimise confounding or for other purposes? I see the analyses are described as descriptive and not intending to make inference, so I am unsure if confounding was part of the modelling strategy or if it was considered unnecessary.

Results:

6. Table 2. Were these unadjusted estimates? For this and later tables / in-text descriptions it would be valuable to clarify whether unadjusted or adjusted associations are being estimated, and the model the statistics come from.

7. Tables: The tables report the OR and 95% CI if results were significant, but omit these results if the findings were non-significant. This may help readers identify the significant results more easily, but it is not aligned with STROBE (item 16) for all results to be reported, and to avoid reporting solely p-values. The non-significant estimates could still be informative, or used in later evidence summaries, and removing them risks introducing reporting bias.

Discussion

8. While the discussion is clear and well written, it was reported in the methods that the intention of the analysis is exploratory and not to infer relationships. However, in the discussion, the results are framed as "to indicate where US is most likely to yield abnormalities, thereby guiding targeted requests and facilitating quicker clinical decisions" - and later sections include generalisation to other populations, and interpreting the findings in terms of decision-making guidance.

This does not seem fully aligned with the framing of the study as exploratory/descriptive. There also may be a risk of confounding - minimising confounding bias was not mentioned in the methods and does not sound like it is part of the approach.

I suggest either reframing the discussion to reflect the exploratory nature of the analysis (e.g. describing correlations within this specific population and using it to suggest more tentative hypotheses regarding clinical practice guidance) or by revising how the methods are described, if the goal is inference.

**Reviewer**	**5**
**Name**	**Sapundzhieva, Tanya**
**Affiliation**	**Medical University of Plovdiv, Internal Diseases**
**Date**	**10-Apr-2026**
**COI**	**None**

The study's findings are clinically meaningful. In routine rheumatology practice, performing ultrasound examinations of all joints is often impractical due to time constraints. Demonstrating that scanning only tender joints is sufficient carries significant practical value and could streamline daily clinical workflows.

A draw-back is turning ultrasound scores into simple positive/negative categories, because it can reduce detail, but the aim of this study was to produce findings that clinicians can easily apply in everyday practice.

Some protocol changes during the study—removing certain joints and adding others later—may have affected how complete the data were for different participants and could have introduced bias when comparing abnormalities across joint sites.

The long recruitment period and the use of several different US machines may also have contributed to variability in image quality, especially since Doppler sensitivity has improved over time.

Another limitation is the absence of formal testing for intra or inter observer reliability.

Several studies report that patients at risk of RA already show ultrasound detected erosions. The authors are encouraged to expand their discussion to address this apparent paradox: how can an erosion, generally understood as a structural lesion resulting from chronic synovial inflammation, arise in individuals who do not exhibit clinical arthritis? Clarifying potential mechanisms—such as subclinical inflammation, mechanical factors, or early immune mediated bone changes—would strengthen the manuscript.

VERSION 2 - AUTHOR RESPONSE

<u>Reviewer: 4</u>
Dr. Kyle Dack, University of Bristol
Comments to the Author:

Statistical review summary:

The paper is well written and the methods/results reporting largely aligns with STROBE guidance. The statistical analyses are all reasonable and implemented well.
However, I have two concerns:

2) First, the results omit some estimates based on statistical significance - this is discouraged because it risks introducing selective reporting bias.

   We thank the reviewer for this observation. In order to provide transparency and avoid selective reporting bias, we have included the full estimates for all analyses (both statistically significant and non-statistically significant associations). This was applicable to tables 3, 4 and supplementary tables S4, S6 and S7.

3) Second, there is a slight misalignment between the introduction and methods where the study is described as exploratory and descriptive, and the discussion where there is clear inference and recommendations for clinical practice. The interpretation therefore seems overly strong or confident, especially because minimizing confounding bias was not described as a part of the methods and appears limited to age and sex.

   We agree with the reviewer that there should be an alignment between the introduction/methods and the discussion. Therefore, we have amended the discussion to make sure that the study findings are not interpreted as recommendations, but rather as exploratory and descriptive. We have also highlighted the cross-sectional design of the study and explained that no causal relationships can be established. Additionally, we have explicitly said that these findings should not be used to guide clinical decision-making without further validation (discussion: page 19, lines 434-439; page 20, lines 455-456; page 22, lines 516-518; page 25, lined 589-596; conclusion: page 26, lines 603-604)

   Regarding the confounding bias, we acknowledge that model adjustment was limited to age and sex. These variables were included based on their association with musculoskeletal symptoms and ultrasound abnormalities. The inclusion of other potential confounders was considered; however, given the low number of events at the joint level, including additional covariates would have risked overfitting and unstable estimates. Therefore, it was agreed that model stability and interpretability would be prioritised. We have clarified this rationale for the covariate adjustment in the methods section and acknowledged in the discussion that residual confounding cannot be excluded (methods: page 14, lines 310-317; discussion: page 24, lines 554-557).

Abstract:

4) There are a few acronyms presented in the abstract before being defined, such as US and anti-CCP. Generally there are quite a lot of acronyms in the abstract which impair readability somewhat, and if any are non-essential (e.g. used infrequently, not standard clinical acronyms) it may be beneficial to reduce them if possible.

   Following the reviewer's advice, we have minimised the acronyms in the abstract in order to improve readability (abstract, pages 3-4).

5) The setting is not reported, and I suggest adding this to the start of the methods.

   Thank you the reviewer for pointing this out. We have amended the methods section in order to provide clarity regarding the study setting (methods section, page 7, lines 146-163).

Introduction:

6) The introduction is clear and summarises the rationale and context for the study nicely. My only suggestion relevant to the introduction (and rest of the paper) relates to that in Comment 1: consider if there are any non-essential acronyms. For example:
   -US - I am unsure if this is a common, standard acronym for ultrasound, but the space saved is minimal, and at a cost of readability.
   -EFSUMB, BME, FT - these and others are only used once in the paper.
   -There is evidence that increased acronym use can impair readability, or increase risk of misinterpretation, so it would be beneficial to apply it to the most frequently used phrases where there will be space saved.
   https://pmc.ncbi.nlm.nih.gov/articles/PMC7556863/,
   https://pmc.ncbi.nlm.nih.gov/articles/PMC7556863/

   Thank you for this helpful comment. As recommended, we have discarded non-essential acronyms (EFSUMB, BME, FT, etc.) and minimised the use of abbreviations, including the replacement of "US" for the full term "ultrasound".

Methods:

7) The data source is described (CCP), is it possible to also comment on the location of the study - is it entirely based in Leeds?

   We have amended the methods section of the manuscript to clarify this: for the primary care part of the CCP study, participants were recruited across England; however, the second part of the study, (which included the baseline assessment used for this cross-sectional analysis) was entirely performed in a dedicated Rheumatology research clinic in Leeds (methods section, page 8, lines 160-163).

8) The description of the variables and statistical analyses is very clear, and decisions are explained/justified well. Regarding the models - it is not clear whether confounding bias was addressed. Models were adjusted for age and sex - was this to minimise confounding or for other purposes? I see the analyses are described as descriptive and not intending to make inference, so I am unsure if confounding was part of the modelling strategy or if it was considered unnecessary.

   We would like to thank the reviewer for this important comment, which we agree it would be important to clarify. Age and sex were included in the models because of their known association with musculoskeletal symptoms and ultrasound abnormalities (aging is associated with the development of wear and tear changes and arthralgia and RA is more prevalent in women). Although the analyses were exploratory and descriptive, we considered that this adjustment was appropriate to reduce confounding. We have explained this in the methods section. We would also like to reiterate that we did not aim to develop causal models; we have acknowledged in the discussion that residual confounding from other factors could persist as a limitation (methods section: page 14, lines 311-317; discussion: page 24, lines 554-557).

Results:

9) Table 2. Were these unadjusted estimates? For this and later tables / in-text descriptions it would be valuable to clarify whether unadjusted or adjusted associations are being estimated, and the model the statistics come from.

   Following the reviewer's recommendation, we have amended all the corresponding tables in the manuscript and in-text descriptions to explicitly say whether estimates were adjusted and from what specific model they originated.

10) Tables: The tables report the OR and 95% CI if results were significant, but omit these results if the findings were non-significant. This may help readers identify the significant results more easily, but it is not aligned with STROBE (item 16) for all results to be reported, and to avoid reporting solely p-values. The non-significant estimates could still be informative, or used in later evidence summaries, and removing them risks introducing reporting bias.

   We would like to thank the reviewer for highlighting this. We have amended the tables of the manuscript (including the supplementary material) to make sure that all results (OR and 95% CI) are reported, regardless of the statistical significance. This was applicable to tables 3, 4 and supplementary tables S4, S6 and S7.

Discussion

11) While the discussion is clear and well written, it was reported in the methods that the intention of the analysis is exploratory and not to infer relationships. However, in the discussion, the results are framed as "to indicate where US is most likely to yield abnormalities, thereby guiding targeted requests and facilitating quicker clinical decisions" - and later sections include generalisation to other populations, and interpreting the findings in terms of decision-making guidance.
This does not seem fully aligned with the framing of the study as exploratory/descriptive. There also may be a risk of confounding - minimising confounding bias was not mentioned in the methods and does not sound like it is part of the approach.
I suggest either reframing the discussion to reflect the exploratory nature of the analysis (e.g. describing correlations within this specific population and using it to suggest more tentative hypotheses regarding clinical practice guidance) or by revising how the methods are described, if the goal is inference.

We appreciate this important comment and agree that the exploratory nature of the study should be aligned with the discussion. Therefore, we have revised the discussion and amended the statements that could have been interpreted as clinical recommendations. Once again, we have highlighted the cross-sectional design of the study and presented the findings as hypothesis-generating associations. We have also clarified that that the results may help future studies assessing whether symptom/sign-based joint selection for ultrasound are efficient, rather than suggesting immediate changes to current clinical practice.

Regarding confounding bias, we have amended the methods section to explicitly say that age and sex were included as potential confounders (due to their known association with musculoskeletal symptoms and ultrasound abnormalities), and that additional covariates were not included in order to avoid overfitting due to the low number of events (methods section, page 14, lines 311-317). We have also acknowledged residual confounding as a limitation in the discussion (discussion: page 24, lines 554-557).

Reviewer: 5
Dr. Tanya Sapundzhieva, Medical University of Plovdiv, University Hospital "Kaspela"
Comments to the Author:

1) The study’s findings are clinically meaningful. In routine rheumatology practice, performing ultrasound examinations of all joints is often impractical due to time constraints. Demonstrating that scanning only tender joints is sufficient carries significant practical value and could streamline daily clinical workflows.

A draw-back is turning ultrasound scores into simple positive/negative categories, because it can reduce detail, but the aim of this study was to produce findings that clinicians can easily apply in everyday practice.
Some protocol changes during the study—removing certain joints and adding others later—may have affected how complete the data were for different participants and could have introduced bias when comparing abnormalities across joint sites.
The long recruitment period and the use of several different US machines may also have contributed to variability in image quality, especially since Doppler sensitivity has improved over time.
Another limitation is the absence of formal testing for intra or inter observer reliability.

We would like to thank the reviewer for this constructive feedback. We agree that the initial version of the discussion could lead to the interpretation of the exploratory findings of this study as "clinical guidance", with the corresponding impact that this would entail. Therefore, we have revised the discussion to ensure that the interpretation of our findings is aligned with the descriptive and hypothesis-generating nature of the study and it is not used to inform clinical decisions directly (discussion, page 19, lines 434-439; page 25, lines 589-592).

We also agree that the limitations of the study should be explained clearly. Therefore, we have expanded the "limitations section" to address the methodological considerations highlighted by the reviewer, and also clarified the potential implications of these limitations for the interpretation of the results (discussion, page 24, lines 552-572).

2) Several studies report that patients at risk of RA already show ultrasound detected erosions. The authors are encouraged to expand their discussion to address this apparent paradox: how can an erosion, generally understood as a structural lesion resulting from chronic synovial inflammation, arise in individuals who do not exhibit clinical arthritis? Clarifying potential mechanisms—such as subclinical inflammation, mechanical factors, or early immune mediated bone changes—would strengthen the manuscript.

Following the reviewer's suggestion, we have also expanded the discussion to address the paradox of erosions being present in individuals without clinical arthritis, including the clarification of potential mechanisms behind the physiopathology or these lesions (discussion, pages 23 and 24, lines 534-550).
